# Biomechanics of the Chick Embryonic Heart Outflow Tract at HH18 Using 4D Optical Coherence Tomography Imaging and Computational Modeling

**DOI:** 10.1371/journal.pone.0040869

**Published:** 2012-07-23

**Authors:** Aiping Liu, Xin Yin, Liang Shi, Peng Li, Kent L. Thornburg, Ruikang Wang, Sandra Rugonyi

**Affiliations:** 1 Department of Biomedical Engineering, University of Wisconsin-Madison, Madison, Wisconsin, United States of America; 2 Department of Biomedical Engineering, Oregon Health and Science University, Portland, Oregon, United States of America; 3 Department of Bioengineering, University of Washington, Seattle, Washington, United States of America; 4 Heart Research Center, Oregon Health and Science University, Portland, Oregon, United States of America; Centrum Wiskunde & Informatica (CWI) & Netherlands Institute for Systems Biology, The Netherlands

## Abstract

During developmental stages, biomechanical stimuli on cardiac cells modulate genetic programs, and deviations from normal stimuli can lead to cardiac defects. Therefore, it is important to characterize normal cardiac biomechanical stimuli during early developmental stages. Using the chicken embryo model of cardiac development, we focused on characterizing biomechanical stimuli on the Hamburger–Hamilton (HH) 18 chick cardiac outflow tract (OFT), the distal portion of the heart from which a large portion of defects observed in humans originate. To characterize biomechanical stimuli in the OFT, we used a combination of *in vivo* optical coherence tomography (OCT) imaging, physiological measurements and computational fluid dynamics (CFD) modeling. We found that, at HH18, the proximal portion of the OFT wall undergoes larger circumferential strains than its distal portion, while the distal portion of the OFT wall undergoes larger wall stresses. Maximal wall shear stresses were generally found on the surface of endocardial cushions, which are protrusions of extracellular matrix onto the OFT lumen that later during development give rise to cardiac septa and valves. The non-uniform spatial and temporal distributions of stresses and strains in the OFT walls provide biomechanical cues to cardiac cells that likely aid in the extensive differential growth and remodeling patterns observed during normal development.

## Introduction

During early developmental stages, blood flow is essential for normal cardiac development [1], [2]. Cardiac cells are subjected to biomechanical stimuli (stresses and strains) that depend on the interaction between blood flow and heart tissues. These biomechanical stimuli modulate cardiac cellular functions and cardiac development. It has been shown that perturbations in blood flow dynamics can lead to structural defects in the heart [1], [3], [4], which occur in about 1% of live births, and are responsible for approximately 10% stillbirths and up to 20% of miscarriages. While cardiac defects can have a genetic origin, it is likely that a large portion of congenital heart defect cases is due to environmental factors, such as alterations of blood flow conditions during early stages of development. To better understand how blood flow conditions affect cardiac development, it is critical to characterize the biomechanical stimuli to which cardiac cells are subjected during development.

Blood flow exerts pressure and shear stresses on cardiac tissues. Equilibrium of forces between the interacting blood flow and cardiac tissue gives rise to internal cardiac wall stresses and cardiac tissue deformation. Endocardial cells, which are endothelial cells that line the heart lumen, are known to be sensitive to shear stresses [1], [5]. Healthy endothelial cells elongate in the direction of flow and perpendicular to the direction of stretch [6], [7], [8], and elongation strongly depends on shear stress conditions. Further, endothelial cells sense and respond to variations in shear stress by mechanosensors, such as the cytoskeleton or monocilia [3], [9]. Therefore endocardial cells are hypothesized to play a fundamental role in cardiac development, with wall shear stresses being a fundamental stimuli modulating cardiac development. Myocardial cells, on the other hand, sense and respond to changes in internal strains and wall stresses [10], [11]. Cardiomyocytes elongate in the direction of maximal stretch [12] and, during development, can alter their rate of maturation and proliferation in response to loading conditions [13], [14], [15]. Proper interaction between endocardial and myocardial cells is also needed to optimize cardiac function and development, and in cardiac adaptation to changing conditions. Since even at early stages of development blood flow is pulsatile, cardiac wall stresses and deformations vary cyclically over time. Further, cardiac cells are constantly adapting to an ever changing biomechanical environment as blood pressure and blood flow continuously increase to satisfy the demands of the growing embryo. Even under these rapidly changing conditions, the biomechanical environment sensed by cardiac cells ultimately determines the fate of the heart. Experimental difficulties in quantifying the biomechanical stimuli to which cells are subjected *in vivo*, however, have hindered progress in understanding how biomechanics modulates cardiac development. In this manuscript, using the chicken embryo heart as a model of cardiac development, we applied an integrative approach to determine biomechanical stimuli to which cardiac cells are subjected during early embryonic developmental stages.

We focused here on the chick cardiac outflow tract (OFT), the distal portion of the heart connecting the primitive ventricle to the arterial system, at an early stage of development, Hamburger–Hamilton (HH) 18 (∼3 days of incubation). At HH18 the heart is an s-shaped tube-like structure with no valves [16], [17], and its OFT wall consists of: (i) a myocardium layer; (ii) an endocardium monolayer, in direct contact with blood flow; and (iii) a cardiac jelly layer, composed of extracellular matrix, and located between the myocardium and endocardium. Localized protrusions of cardiac jelly form endocardial cushions, which act as primitive valves that facilitate lumen closure [18]. The OFT is an important heart segment that undergoes extensive morphogenesis and eventually develops into aortic and pulmonary outlets and gives rise to semilunar valves [19], [20]. The morphogenesis of the OFT is sensitive to biomechanical stimuli [21], [22], and a large portion of congenital heart defects originate in the OFT [22].

To quantify biomechanical stimuli in the OFT, we used a combination of cardiac imaging, physiological measurements (blood pressure and blood flow velocities), and computational fluid dynamics (CFD) modeling. Current state-of-the-art optical coherence tomography (OCT), a high resolution (2–20 µm) non-invasive (non-contact) tomographic imaging technique [23], [24], allows studying heart dynamics in 4D (3D over time) [25], [26]. Using OCT imaging, we first characterized the *in vivo* motion of the OFT wall over the cardiac cycle, and cardiac wall strains. Using the dynamic geometry of the OFT wall, and blood pressure measurements from the developing heart, we estimated myocardial wall stresses and developed dynamic image-based (subject-specific) CFD models of the developing heart OFT to quantify *in* *vivo* blood flow patterns and wall shear stresses over the cardiac cycle. The combination of OCT imaging and CFD modeling provided a detailed characterization of the *in vivo* dynamic biomechanical stimuli to which cardiac cells are exposed during the cardiac cycle in the OFT of HH18 chicken embryos. Results from this paper could lay the foundation for future studies to establish the role of biomechanics on cardiac development, and in particular on detrimental cardiac adaptations that lead to congenital heart disease.

## Materials and Methods

### Chick Embryo Preparation

Fertilized White Leghorn eggs were incubated, blunt end up, at 38°C and 80% humidity to stage HH18 in a positive flow incubator with self-filling reservoir. Before imaging or pressure measurement, a small window was opened on the egg shell at the air sac end and the underlying membrane was removed to expose the embryo heart. During embryo manipulation, the temperature of the chick embryo was maintained at 37.5±0.5°C within a custom-made warming chamber using a temperature controller. Keeping the embryos warm, at approximately constant temperature, is important because temperature changes affect cardiovascular function.

### 4D OCT Imaging and Reconstruction

We used a spectral-domain OCT system customized to image the structure and blood flow of the chick OFT *in ovo* [27], [28]. The system used a superluminescent diode broadband light source with full-width-half-maximum of 56 nm centered at 1310 nm (Denselight, Singapore), which yielded an axial spatial resolution of 10 µm and a lateral spatial resolution of 16 µm. The system further consisted of a 1024 element infrared InGaAs line-scan camera with 14 bit digital depth and a maximum line-scan rate of 47 kHz, which allowed acquisition of 2D images (B-mode) of 512×256 pixels (256 A-scans) at 140 frames per second (fps).

To capture the dynamics of the fast beating embryonic heart (typical heart rate is about 2.5 Hz at HH18), we used our previously developed 4D imaging and reconstruction procedures [26]. Briefly, we acquired 2D image sequences at a rate of 140 fps over 4–5 cardiac cycles on fixed imaging planes. To image the entire OFT, 2D image sequences were acquired on consecutive, adjacent imaging planes that were 7.5 µm apart, spanning the entire length of the OFT. Imaging the entire OFT took ∼20 min per embryo. Because acquisition of image sequences was not triggered by a cardiac signal, the sequences were not synchronized (i.e. sequences started at different phases of the cardiac cycle). To obtain 4D images of the OFT, we first synchronized the acquired image sequences using spatial similarities between neighboring image sequences along the heart, taking also into account phase lags due to the peristaltic-like motion of the OFT walls [26]. Then we reconstructed the 3D geometry of the OFT at discrete time points (phases) over the cardiac cycle. Resulting 4D data sets consisted of 196 3D volume images that span the OFT motion over the cardiac cycle, with images slightly resized (to 160 frames along the OFT with each frame consisting of 180×265 pixels) so that each voxel was 5 µm×5 µm×5 µm.

### 4D Image Processing

To analyze the dynamics of wall motion and blood flow in the OFT from 4D OCT images, we developed image-analysis algorithms using Matlab2009a (The MathWorks, Inc. Natick, MA).

#### a) Geometry and wall motion

To quantify the geometry and motion of the OFT wall, we used a custom-made hybrid segmentation algorithm that we developed and implemented for 3D analyses. The algorithm, which combines an optical flow technique [29] with active deformable (contour and surface) models [30], [31], enabled automatic delineation (segmentation) of myocardium and endocardium surfaces over time, from cross-sectional image sequences (2D + time), or over space, from 3D images (3D volume data).

**Figure 1:**
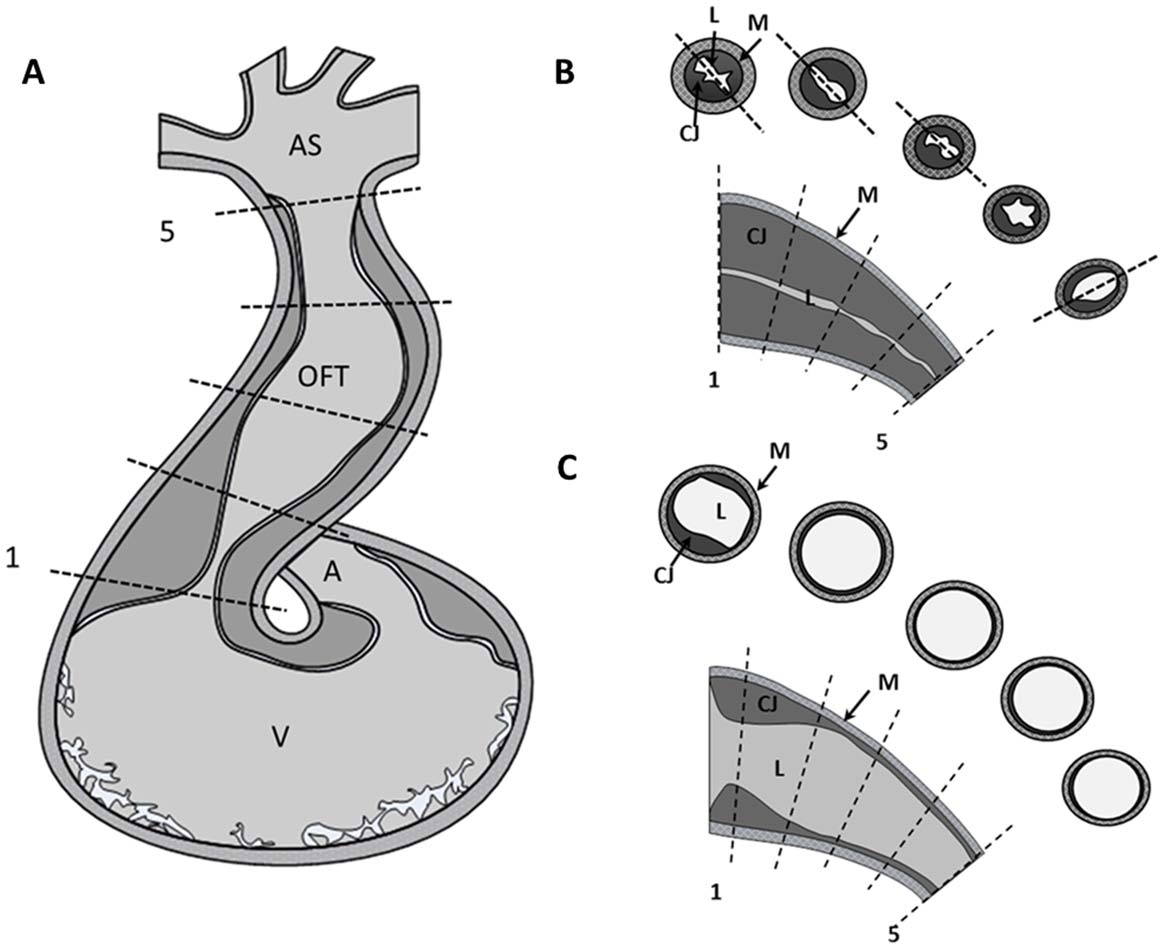
Morphology of HH18 chick outflow tract (OFT). (A) Sketch of the HH18 developing heart showing the approximate location of cross-sectional planes analyzed from 4D OCT images. (B) and (C) Sketch of OFT wall configuration during myocardial contraction and maximal myocardial expansion, respectively, along longitudinal and cross-sectional cuts of the OFT. Lines 1 to 5 along the longitudinal sections show the location and approximate orientation of the planes from which cross-sectional image sequences where extracted from 4D OCT images for analysis; depicted cross-sections correspond to each of the 5 lines along the OFT (see also video S1). The dashed lines in cross-sectional sketches from panel (A) show the elongation of the lumen along the cardiac cushions. A, atrium; V, ventricle; AS, aortic sac; M, myocardium; L, lumen; CJ, cardiac jelly.

For each reconstructed 4D cardiac image, we first segmented the myocardium layer when the OFT was most constricted and calculated the tubular-heart OFT centerline. We then selected 5 evenly spaced points along the centerline and corresponding cross-sectional planes centered at the selected points, and perpendicular to the centerline tangent (see Figure 1A, planes 1 to 5 from proximal to distal OFT regions; see also Video S1). We then generated 2D image sequences that showed the motion of the OFT over the cardiac cycle at each of the selected cross-sectional planes (which were fixed in space). Finally, from cross-sectional images we delineated the contours of the OFT myocardium (inner and outer contours) and endocardium from the image sequences, frame-by-frame (Figure S1), and calculated the boundary perimeters, and the areas enclosed by the contours.

To further quantify OFT wall motion, we extracted M-mode images from cross-sectional image sequences, along a line perpendicular to the cardiac cushion surface. M-mode images, which show intensity along a line (vertical direction) over time (horizontal direction), allow easy visualization of cardiac motion over cardiac cycles and quantification of OFT lumen closure and opening.

#### b) Wall strains and area shortening fraction

Circumferential strains, ε_θ_, in the OFT myocardium and endocardium were estimated using the segmented contour perimeter C,
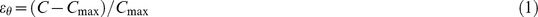
(1)where C_max_ is the maximum value of the perimeter over the cardiac cycle, and was chosen here as the reference for strain calculations.

Variations in the areas enclosed by the inner myocardium and the endocardium contours, as well as variations in the area of the cardiac jelly (calculated as the difference between the areas enclosed by the myocardium and the endocardium), were quantified as area shortening fractions (ASF),
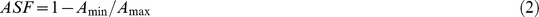
(2)


#### c) Blood flow measurement using OCT

The unique advantage of spectrum domain OCT is that during imaging both structural and flow data within the OFT were obtained simultaneously. Phase images were obtained by calculating the phase differences Δϕ between two adjacent A-scans in a B-scan. This phase difference is introduced by the movement of tissue and cells, such as the motion of blood, and can be converted to Doppler velocity [28],
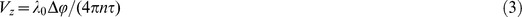
(3)where V_z_ (the Doppler velocity) is the projection of the velocity vector in the direction of the light beam, λ_0_ is the central wavelength (1310 nm), *n* is the refractive index of tissue (∼1.3), and τ is the time difference between two adjacent A-scans (∼21 µs). The magnitude of the flow velocity vector, *V*, can then be obtained by correcting V_z_ with the Doppler angle, θ, the angle between the light beam direction and the direction of blood flow, using the equation:



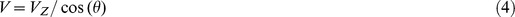
(4)To further quantify blood flow dynamics within the OFT over the cardiac cycle, we extracted M-phase images, which are similar to M-mode images but show phase instead of structural motion. M-phase images showed the phase Δϕ (−π, π) in the vertical direction extracted from a line in the acquired image sequence, over time (horizontal direction). M-phase images were used to visualize flow over the cardiac cycle, and together with corresponding M-mode images, were used to determine the relationship between cardiac wall motion and flow in the OFT.

### Pressure Measurements and Myocardial Wall Stress

We used a servo-null micro-pressure system (Model 5A-LN, Instrumentation for Physiology and Medicine, San Diego, CA) to measure blood pressures in normal chick embryonic hearts at HH18. Pressure data were collected from the heart ventricle and aortic sac, which are located immediately upstream and downstream of the OFT, respectively, following standard procedures [32]. Pressure traces were sampled at 100 Hz over at least 10 cardiac cycles in the ventricle and in the aortic sac.

To approximate the circumferential wall stress σ_θ_ in the myocardium, we used the Laplace law,
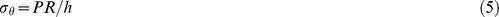
(5)where *P* is the intracardiac blood pressure, *R* is the inner radius of the myocardium layer (assuming a circular myocardium shape), and *h* is the average wall thickness of the myocardium. Geometric parameters were quantified from OCT images.

### Computational Fluid Dynamics Modeling and Wall Shear Stress

To quantify 3D blood flow dynamics and wall shear stresses over the cardiac cycle, we developed a subject-specific computational fluid dynamics (CFD) model of the chick OFT. A detailed description of the CFD model development and assumptions were given elsewhere [33]. Briefly, our CFD model considered the 3D curvature and non-circular cross-sections of the OFT lumen, as well as the heart wall motion and pulsatile pressures. Given the low hematocrit of blood at early stages of development [34], blood was modeled as Newtonian, and blood flow was calculated using the transient Navier-Stokes equations. The lumen geometry and wall motion of the CFD model were obtained from 4D OCT imaging data of a ‘representative’ HH18 embryo. The lumen was assumed to have an elliptical shape, fitted from lumen segmentations of OCT images at the 5 selected cross-sectional planes (see Figures 1A and 1B; and also Figures S1C to S1F). To model the large variations in the shape and area of the lumen, which take place over the cardiac cycle and along the OFT, ellipses at each selected cross-section changed size and shape. This change was implemented as a displacement field imposed on OFT walls that varied over time and space. The displacement was calculated from the elliptical fitted shapes, and was imposed on the model surface representing the OFT lumen-wall interface, to simulate the motion of the cardiac OFT wall. Representative ventricular and aortic sac pressures, obtained from *in vivo* measurements, were imposed uniformly on the inlet and outlet surfaces, respectively, as normal traction boundary conditions after correction for the approximate distance between the ventricle and aortic sac to the modeled OFT inlet and outlet, respectively. We used pressure as boundary conditions in our model because pressure measurements were available, while we did not have accurate spatial distributions of inlet or outlet OFT blood flow velocities. This, however, allowed us to use subject-specific Doppler OCT centerline blood flow velocities to validate the model. The wall motion extracted from OCT images and the pressure measurements (used as boundary conditions in the CFD model) were from different embryos because of limitations in performing both imaging and pressure measurements procedures on the same embryo. Thus, phase relationships between blood pressure and motions were estimated.

In contrast to the previously published CFD model [33], in the OFT model presented here, the lumen area and shape were more accurately extracted. This is because our 3D segmentation algorithms for lumen-wall interface extraction were significantly improved by carefully optimizing the values of segmentation parameters and procedures to extract the heart centerline. This resulted in a better orientation of cross-sectional planes, and more accurate delineation of the lumen-wall interface. Further, rather than simulating the whole cycle, we only simulated half of the cardiac cycle (0.43 T to 0.93 T, where T is the period of the cycle). This is because analyses of Doppler OCT velocity measurements in the OFT of HH18 chick embryos presented here revealed negligible flow in the OFT for ∼0.5T, when the OFT walls were contracted. Overall the CFD model presented here has been improved from that of the previous paper [33], and its results complement the experimental data in the OFT of chicken embryos, allowing fully characterization of hemodynamic conditions at HH18.

The CFD model of the OFT was implemented using finite element methods in the software ADINA (ADINA R & D, Inc. Watertown, MA). Simulation results were visualized and further post-processed using the software EnSight (CEI, Apex, NC). Specifically we used 3D 4-node tetrahedral flow-condition-based interpolation (FCBI) elements to discretize the OFT lumen; and mesh independent solutions were achieved with 440,240 elements. Time step independent results were achieved using 200 steps (T = 370 ms; Δt = 0.925 ms) to capture transient flow phenomena in the OFT. Using this CFD model, we quantified the temporal and spatial variation of blood flow velocities within the OFT and wall shear stresses on the endocardium. Wall shear stress was visualized using EnSight, as the product of the flow viscosity (µ = 3×10^−3^ kg/(m s)) and the gradient of velocity in the direction normal to the OFT wall. Centerline blood flow velocities obtained with our subject-specific CFD model were compared to Doppler OCT velocity measurements at corresponding centerline locations, from the same representative chick embryo used for the model development.

## Results

To characterize the biomechanical stimuli to which chick embryonic OFT cardiac cells are subjected at HH18, we employed an integrative approach that uses OCT imaging, blood pressure measurements, and CFD modeling. OCT imaging allowed extraction of the cardiac geometry and quantification of cardiac tissue dynamics (displacements, strains and rates of strain) as well as blood flow dynamics (from Doppler OCT). Measurements of blood pressure together with cardiac tissue dynamics, allowed estimation of cardiac wall stresses. Doppler OCT measurements, complemented with CFD modeling, allowed characterization of blood flow dynamics and wall shear stresses. This characterization of OFT tissue strain and stresses is critically needed to fully understand the role of biomechanics on cardiac development.

### Characterization of OFT Wall Dynamics

We imaged and analyzed the OFTs from seven HH18 chick embryos. OCT images showed the microstructure of the OFT wall (see Video S1 and Figure S1). The myocardium and cardiac jelly layers were readily distinguished; the endocardium layer, however, was not distinguishable from the lumen blood due to their similar refractive index. Since the endocardium is a monolayer of endocardial cells, we initially neglected its thickness and assumed that the endocardium and wall-lumen interface surfaces were coincident.

4D image reconstructions of the HH18 heart showed the characteristic peristaltic-like dynamic motion of the OFT wall. When the OFT wall expanded, blood ejected from the ventricle to the arterial circulation. After blood ejection, the OFT wall contracted, and the endocardial cushions came in contact with each other, closing the lumen proximally to distally to prevent backflow.

To further quantify OFT wall motion, we analyzed the motion of the myocardium, cardiac jelly, and endocardium layers using 2D image sequences extracted from 5 cross-sectional planes along the OFT (planes 1 to 5, Figure 1). For these cross-sectional image sequences, the frame planes were fixed in space, and approximately perpendicular to the OFT centerline. Results are illustrated for a ‘representative’ embryo, which was also chosen for the subject-specific CFD modeling, and also shown as average values and standard deviations for the group (n = 7; Table 1).

**Table 1:** Summary of measured OFT biomechanical parameters at the 5 selected cross-sections (L1 to L5).

Plane	1	2	3	4	5
**Myocardium**					
λ_max_	1.27±0.06	1.21±0.08	1.2±0.1	1.26±0.07	1.19±0.09
λ_min_	1.06±0.02	1.06±0.03	1.08±0.05	1.11±0.06	1.09±0.07
R_max_ [mm]	0.21±0.02	0.20±0.02	0.19±0.02	0.17±0.03	0.15±0.01
R_min_ [mm]	0.14±0.01	0.13±0.01	0.12±0.02	0.11±0.01	0.12±0.01
ΔR [mm]	0.07±0.01	0.07±0.01	0.073±0.009	0.06±0.01	0.035±0.008
Peak contraction ε_θ_	0.33±0.04	0.36±0.04	0.39±0.03	0.36±0.03	0.23±0.05
**Cardiac jelly**					
A_max_ [mm^2^]	0.06±0.02	0.05±0.01	0.03±0.01	0.027±0.008	0.026±0.006
A_min_ [mm^2^]	0.039±0.008	0.028±0.008	0.021±0.007	0.013±0.005	0.014±0.004
ΔA [mm^2^]	0.021±0.009	0.014±0.006	0.010±0.005	0.009±0.005	0.009±0.004
ASF	0.33±0.07	0.31±0.07	0.29±0.09	0.3±0.1	0.4±0.1
**Endocardium**					
C_max_ [mm]	1.07±0.05	1.1±0.1	1.1±0.1	0.9±0.2	0.83±0.08
C_min_ [mm]	0.68±0.08	0.59±0.07	0.56±0.09	0.47±0.08	0.58±0.08
Peak contraction ε_θ_	0.37±0.07	0.44±0.05	0.47±0.05	0.49±0.03	0.3±0.1
**Lumen**					
A_max_ [mm^2^]	0.09±0.01	0.09±0.02	0.09±0.02	0.07±0.02	0.05±0.01
A_min_ [mm^2^]	0.022±0.004	0.019±0.004	0.017±0.004	0.014±0.005	0.022±0.005
ΔA [mm^2^]	0.07±0.01	0.07±0.02	0.07±0.02	0.06±0.02	0.03±0.01
ASF	0.75±0.05	0.79±0.04	0.81±0.03	0.79±0.03	0.60±0.08
λ_max_	2.7±0.4	2.5±0.4	2.1±0.4	1.7±0.3	1.6±0.2
λ_min_	1.2±0.1	1.14±0.08	1.12±0.09	1.12±0.06	1.1±0.1

Data presented as mean±standard deviation. λ, shape factor; R, radius; ΔR, radius change over the cardiac cycle; ε_θ_, circumferential strain; A, area; ΔA, area change over the cardiac cycle; ASF, area shortening fraction.

#### a) Myocardium

**Figure 2:**
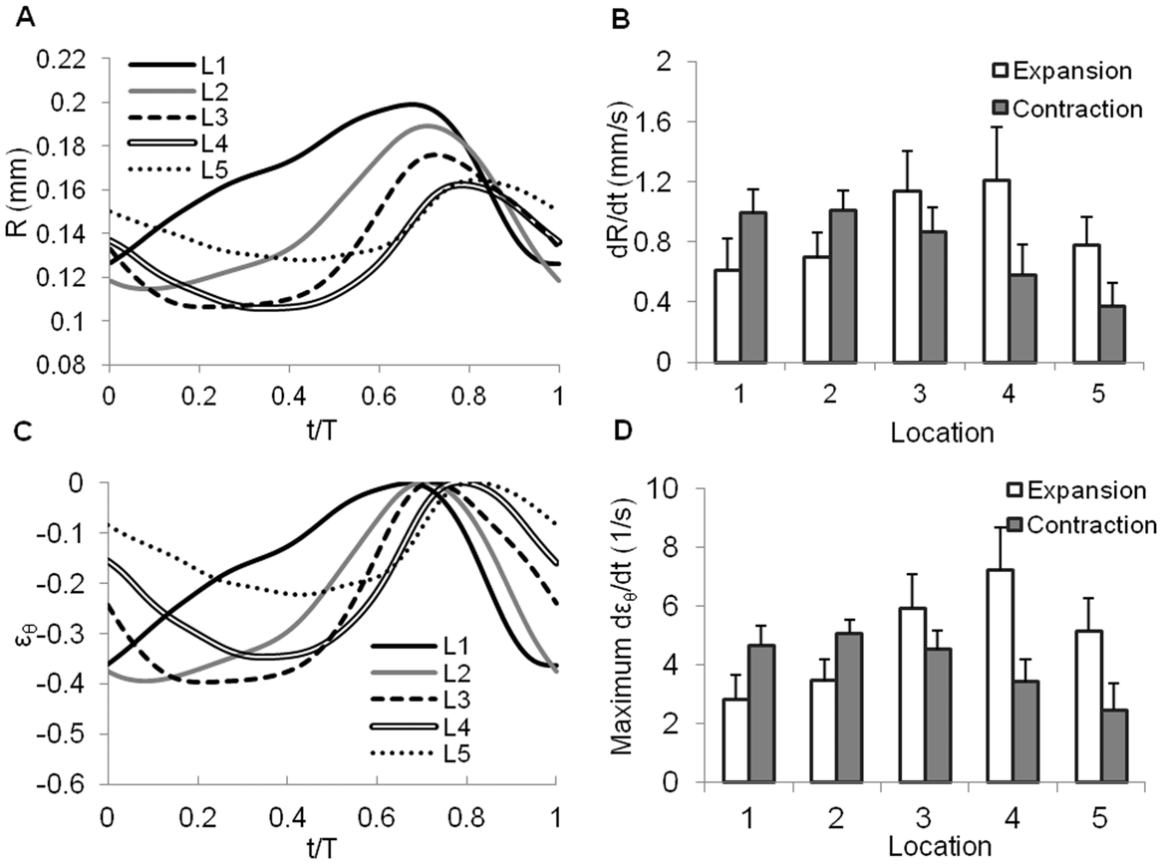
Motion of the OFT myocardium at the 5 selected cross-sections along the OFT. (A) Variation of internal myocardial radius (R) over time. (B) Maximal absolute radial velocity (dR/dt) of the internal myocardial boundary during expansion and contraction. (C) Myocardial circumferential strain (ε_θ_). (D) Maximal circumferential strain rate (dε_θ_/dt) of the myocardium during expansion and contraction. Time (t) was normalized to the cardiac cycle (T). L1 to L5 refer to the 5 cross-sectional planes selected for analysis (see Figure 1A, lines 1 to 5).

In cross-sectional images, the myocardium had a slight ellipse-like shape. The ratio of the length of the major and minor axes, the shape factor λ, was around 1 (circular shape) when the OFT myocardium was most expanded; and 1.3 when the myocardium was most constricted (Table 1); hence the myocardium was approximately circular over the cardiac cycle. For simplicity, myocardial dimensional changes were characterized by an effective myocardial radius, 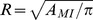, where *A_MI_* was the area enclosed by the segmented inner contour of the myocardium. Plots of the effective radius R quantified from the selected cross-sections over time showed a peristaltic-like motion and tapering of the myocardial wall (Figure 2A), with the amplitude of the myocardial motion decreasing proximally to distally (see also Table 1). We further calculated the myocardial radial velocity, dR/dt, and compared the maximum rates of myocardial expansion and contraction along the OFT (Figure 2B). We found that from planes 1 to 5 maximal expansion velocity generally increased and then decreased, while maximal contraction velocity decreased. Interestingly, while in the proximal region of the OFT contraction velocities were on average slightly larger than expansion velocities, this behavior reversed distally (Figure 2B).

Circumferential strains, calculated using Eq. 1, varied over the cardiac cycle, as expected. Peak contraction strains were similar at planes 1 to 4, but dropped significantly at plane 5 (Figures 2C). Further, circumferential strain rates during expansion first increased from the OFT inlet, reached a maximum, and then decreased further distally; and during contraction decreased distally (Figure 2D). Together these data suggest that myocardial contraction diminishes towards the distal portion of the OFT.

From OCT images, we also observed that the OFT wall stretched longitudinally (Video S1). This longitudinal motion decreased distally towards the aortic sac, which seemed to be tethered. Proximally, however, the contraction and expansion of the ventricle imposed an apparent motion on the OFT wall. By tracking an anatomical landmark, the sharp curvature that the cardiac wall forms at the intersection between the OFT and the ventricle (see Video S1), we found the OFT wall moved longitudinally about 140 µm over the cardiac cycle. Since the length of the OFT is about 600 µm, this motion corresponded to about 20% stretch in the longitudinal direction.

#### b) Cardiac jelly

The cardiac jelly was generally divided into two endocardial cushions (Figure 1A). The orientation of the cushions spiraled along the OFT, especially between cross-sectional planes 3 and 5, with the orientation at plane 4 ambiguous, since the lumen had a star-like shape at contraction there (5/7 embryos). This is consistent with the described OFT of HH21 embryos [20] that have two pairs of spirally distributed cardiac cushions: a proximal cushion pair and a distal cushion pair. OCT images indicated that the proximal cushion pair extends from plane 1 to plane 4, and the distal cushion pair starts around plane 4 and extends to plane 5.

**Figure 3:**
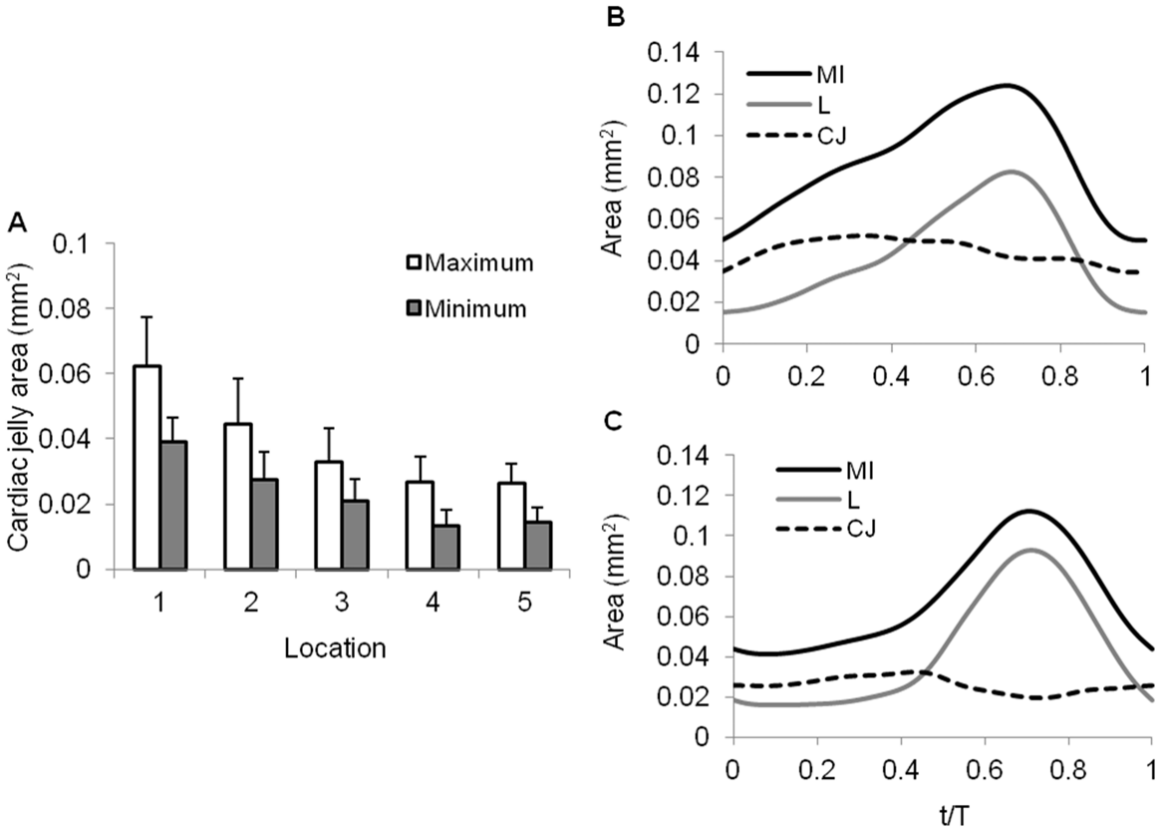
Area of the OFT cardiac jelly layer quantified from cross-sectional images. (A) Maximal and minimal areas of the cardiac jelly at the 5 selected cross-sections along the OFT (L1 to L5, see Figure 1A). Changes in cardiac jelly area over time, along with corresponding changes in myocardial and lumen areas at (B) L1 and (C) L2.

The area of the cardiac jelly decreased distally (Figure 3A and Table 1). Within cross-sectional planes, maximum cardiac jelly areas were consistently found when the contracted OFT wall started to expand, whereas minimum cardiac jelly areas occurred during OFT contraction phases (Figures 3B and 3C).

#### c) Endocardium and lumen

**Figure 4:**
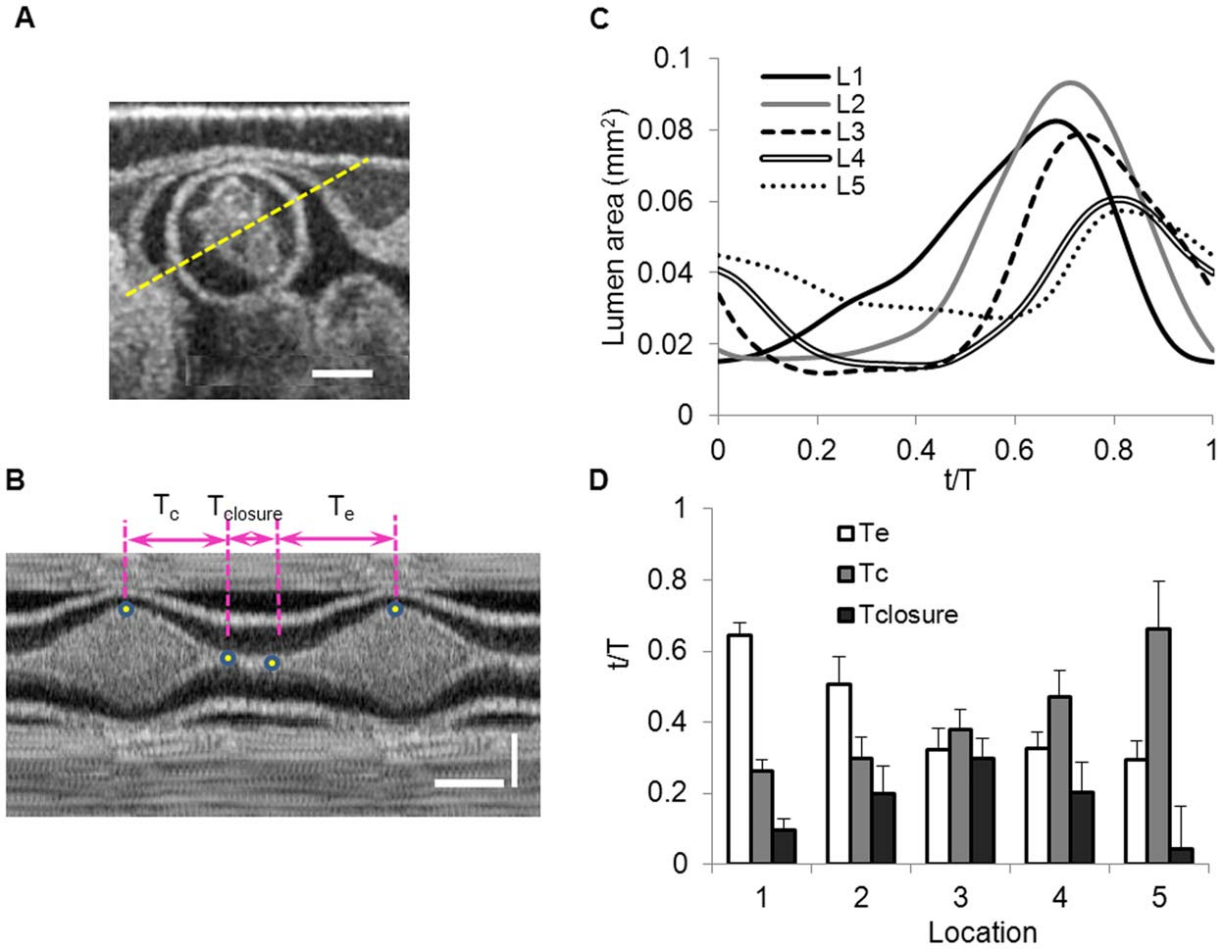
Quantification of OFT lumen behavior. (A) OCT cross-sectional image of the OFT depicting the irregular shape of the lumen at mid-myocardial contraction (scale bar = 200 µm). (B) M-mode image obtained from cross-sectional image sequences along the yellow dashed line in (A), depicting the quantification of the time during which OFT walls were contracting (T_c_), closed (T_closure_), or expanding (T_e_). Horizontal scale bar = 100 ms and vertical scale bar = 200 µm. (C) Calculated lumen area over the cardiac cycle for the 5 selected cross-sectional planes (L1 to L5, see Figure 1A). (D) Fractional time over one cardiac cycle during which OFT walls were expanding (T_e_), contracting (T_c_) and closed (T_closure_). t, time; T, cardiac period. Note that at the cross-sectional plane 5 (L5) only 5 out of 7 embryos showed lumen closure.

The OFT lumen, and therefore the endocardium layer, exhibited large shape changes over the cardiac cycle: from a slit-like shape during contraction (where slit ‘branches’ correspond to endocardial folds [35]), to an almost circular shape during maximal myocardial expansion (Figure 1B, see also Video S1). We used area to describe the cyclic changes of the OFT lumen (Figure 4C and Table 1). Lumen area increased during myocardial expansion and decreased during contraction. Maximal lumen area decreased proximally to distally, following the tapering of the OFT myocardial wall, but with a slight increase in lumen area approximately in the middle part of the OFT, due to the distribution of cushions. The lumen was approximately elliptical in shape, except when lumen area was minimal or nearly minimal, and endocardium ‘folds’ were observed.

Since the endocardium and the lumen cannot be distinguished from OCT images, we conjectured that during contraction the lumen was fully closed, and the calculated area corresponded to the area occupied by the endocardium. Using the segmented length of the endocardium boundary, and the area enclosed by it, we estimated the thickness of the endocardium layer. For cross-sectional planes 1 to 4, the calculated thickness was 18±4 µm, which is consistent with the thickness of a monolayer of endocardial cells. However, for cross-sectional plane 5 the estimated thickness was 31±3 µm (5/7 embryos), suggesting that the lumen did not fully closed in distal regions of the OFT. This is also consistent with less contraction and motion of the myocardium in the distal OFT region.

**Figure 5:**
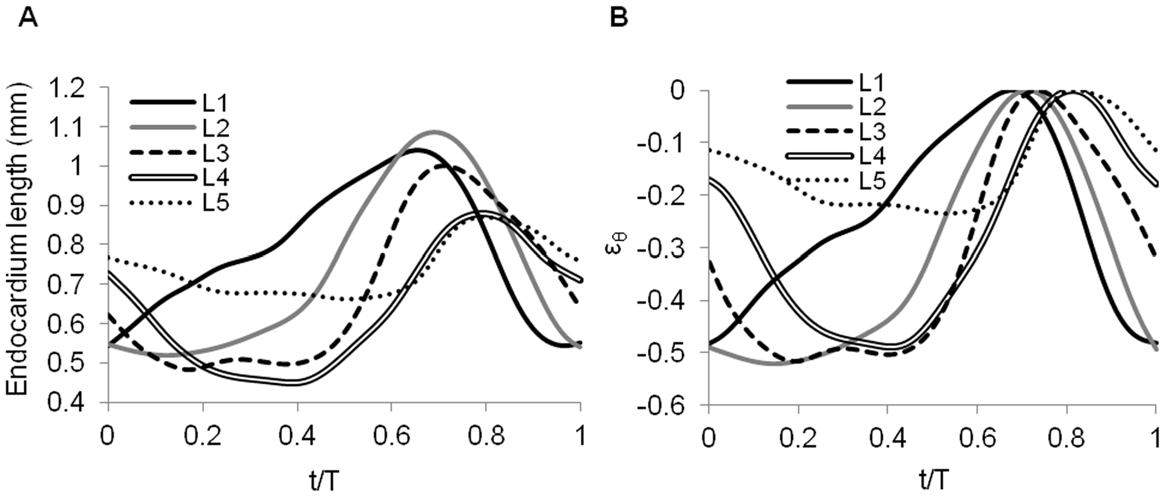
Changes in endocardium length and strain over the cardiac cycle. Temporal variations of (A) the length of the endocardium and (B) circumferential strains (ε_θ_) in the endocardium at the 5 selected OFT cross-sections (L1 to L5, see Figure 1A).

**Figure 6:**
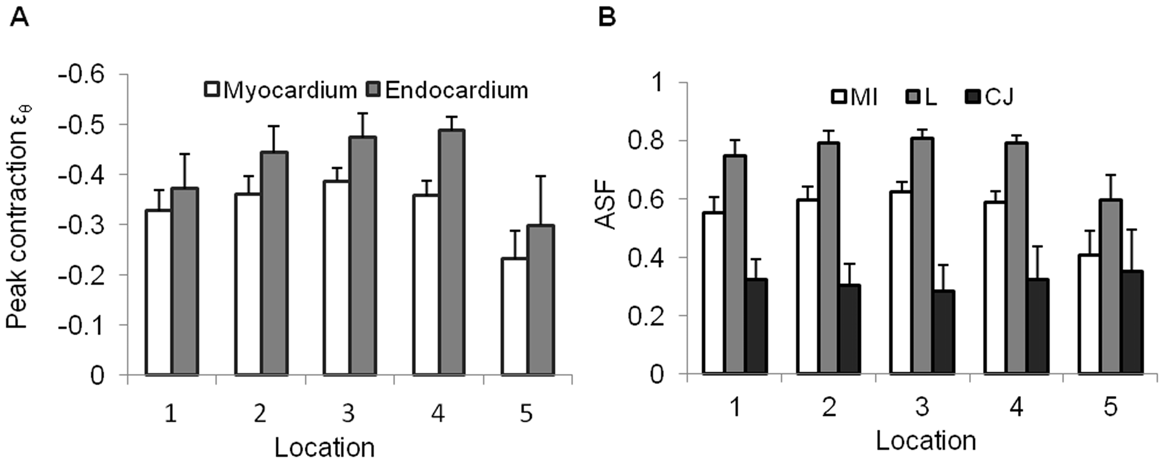
Comparison of wall-motion metrics at the 5 selected OFT cross-sectional planes. (A) Peak contraction circumferential strain (ε_θ_), and (B) area shortening fraction (ASF). MI: myocardium, L: lumen, CJ: cardiac jelly.

The endocardium is generally considered as a passive layer: its length shortens as a result of active myocardial contraction and expands due to myocardial relaxation and blood filling in the lumen (Figures 5A, 2A and 4C). Variations of circumferential strain in the endocardium over the cardiac cycle, measured as differences in length with respect to the maximum length of the segmented endocardium curve, Eq. 1, were similar to variations of circumferential myocardial strains (see Figures 5B and 2C). Endocardial strains, however, were larger than myocardial strains (Figure 6A).

To better characterize OFT wall motion at HH18, we analyzed M-mode images extracted along a line that approximately aligned with the minor axis of the elliptical lumen (Figures 4A and 4B). M-mode images revealed that the fraction of the time during which the OFT walls expanded (T_e_) decreased towards the distal end of the OFT; while wall contraction time (T_c_) increased towards the distal end of the OFT (Figure 4D). The time span over which the OFT lumen area was minimal (T_closure_), was highest at the middle of the OFT (plane 3) and decreased proximally and distally.

### Changes in Intracardiac Pressures and Myocardial Wall Stresses

**Figure 7:**
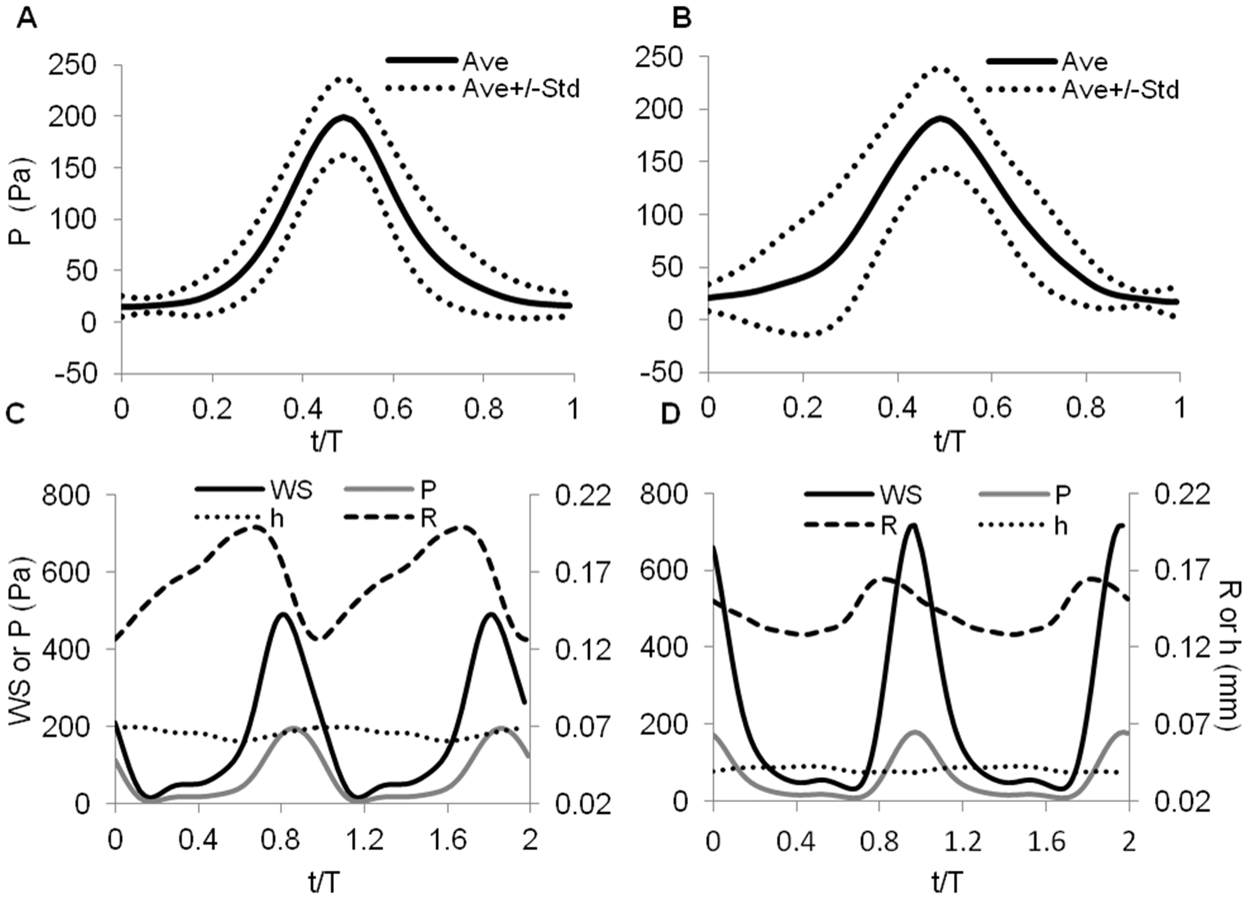
Intracardiac blood pressure and myocardium wall stress. Measured blood pressures in (A) the ventricle and (B) the aortic sac of HH18 chick embryos. Solid line shows averages and dotted lines standard deviations for the aggregate data. Note that in these plots (A and B) the peaks of the blood pressure measurements are arbitrarily aligned at t/T = 0.5. Estimated myocardial wall stress at (C) OFT inlet (plane L1), and (D) OFT outlet (plane L5). (C) and (D) also show for reference corresponding blood pressures, internal myocardial radius and myocardial wall thickness used to calculate wall stress. WS, wall stress; P, intracardiac blood pressure; R, radius of the internal myocardium boundary; h, myocardial wall thickness.

Ventricular pressures (n = 29) and aortic sac pressures (n = 9) were measured in the ventricle and aortic sac, located immediately upstream and downstream of the OFT, respectively (Figures 7A and 7B). Ventricular pressure data was consistent with previously reported blood pressures from HH18 embryos [36], [37], [38]. The curve of the aortic sac pressure over time was similar to that of the ventricular pressure, but the peak ventricular pressure was in average slightly higher than the peak aortic sac pressure (196±37 Pa vs. 180±35 Pa, respectively).

To estimate wall stress in the OFT myocardium, we used representative ventricular and aortic sac pressures. We then approximated myocardial wall stress at cross-sectional planes 1 and 5 using the Laplace law (Figures 7C and 7D). Our results showed that peak wall stress on the OFT was larger distally (plane 5) than proximally (plane 1). These results hold even if different phases are assumed among cardiac tissue motion and blood pressure.

### Interaction between Wall Motion and Blood Flow

To characterize the interaction between cardiac wall motion and blood flow dynamics within the OFT, we analyzed OCT structural and Doppler velocity images from acquired 2D image sequences of the representative chick embryo. Since the image signal degrades towards the OFT outlet (which is deeper within the egg), due to signal adsorption and washout effect [28], we chose cross-sectional image sequences near the OFT inlet, which approximately included the centerline points at the cross-sectional planes 1 and 2. Note that the image plane and the OFT cross-sectional planes extracted from the 4D image data were not coincident; the rotation of the cross-sectional plane with respect to the imaging plane determined the Doppler angle.

**Figure 8:**
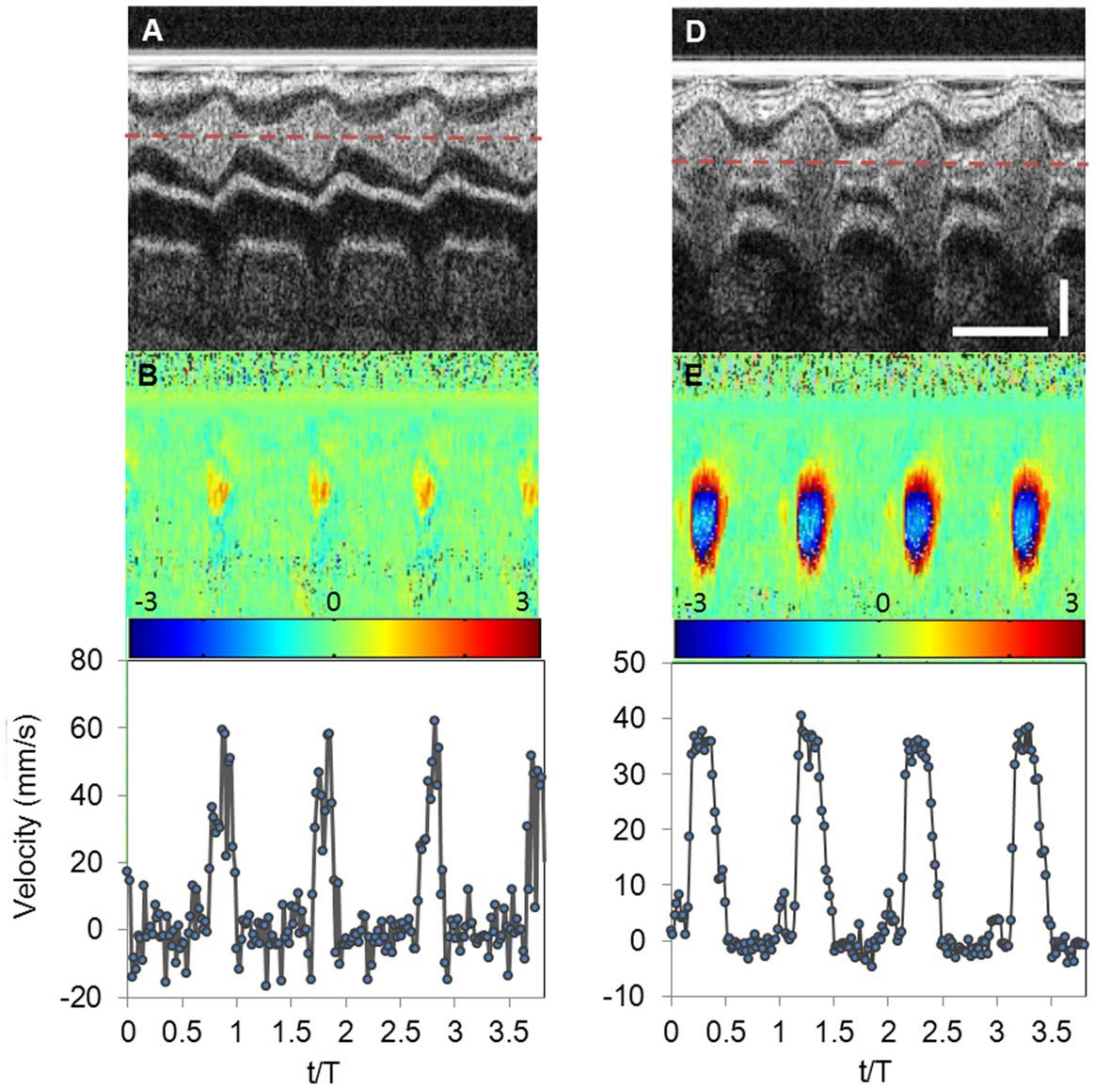
Simultaneous structural and phase OCT data analyses. Left (A, B, C) and right (D, E, F) panels show data extracted from an image sequence that approximately contains the OFT centerline point of cross-sectional plane 1, and cross-sectional plane 2, respectively (see Figure 1A). (A) and (D) M-mode structural images, from a line along the OFT image sequence that approximately cut the OFT in half and contained the centerline point. (B) and (E) Corresponding M-phase images (phase angle expressed in radians). (C) and (F) Magnitude of blood velocity vector over the cardiac cycles, extracted along the dotted line in (A) and (D). t: time; T: cardiac cycle period. In (D), horizontal scale bar = 500 ms, and vertical scale bar = 200 µm.

To facilitate the analysis of simultaneous wall motion and flow, we extracted M-mode and M-phase images (see Figure 8) from corresponding structural and phase image sequences along a vertical line that approximately cut the OFT cross-section in two halves. In the M-phase image, we observed phase wrapping (see Figure 8E), which occurred when blood flow was fast and Δϕ exceeded the value of π and suddenly jumped to −π [28], and was evident as a negative phase (blue) enclosed by positive phase (red). Phase wrapping is undesirable, as it involves inaccuracies in flow measurements. Nevertheless, the combination of M-mode and M-phase images showed that blood flow initiated when the OFT walls started to expand and ended when the OFT walls contracted and the lumen was almost closed. Further, we found that there was no significant blood flow for approximately half of the cardiac cycle.

To quantify time variation of centerline blood flow velocities, we chose a line from the M-mode images that remained in the lumen at all times (except when the OFT was fully closed), and extracted the phase along that line, which corresponded to Δϕ of a point approximately located in the center of the lumen over time. Phase un-wrapping was performed according to established procedures. Then, using Eqs. 3 and 4, and the Doppler angle (84° for cross-sectional plane 1; and 58.4° for plane 2) derived from 4D structural information [28], we estimated the centerline blood flow velocities *V* at the cross-sectional planes 1 and 2 (Figures 8C and 8F). The centerline blood flow velocities exhibited a fast acceleration followed by a fast deceleration at both planes, corresponding to the fast opening and closing of the lumen and myocardium over time. Peak blood flow occurred when the OFT wall was fully expanded. The value of peak blood flow velocity was larger at the cross-sectional plane 1 than at plane 2, likely because the lumen area of plane 2 was larger than the lumen area of plane 1 (see Figures 8A and 8D), and due to wrapping inaccuracies.

### Blood Flow Dynamics and Wall Shear Stresses

**Figure 9:**
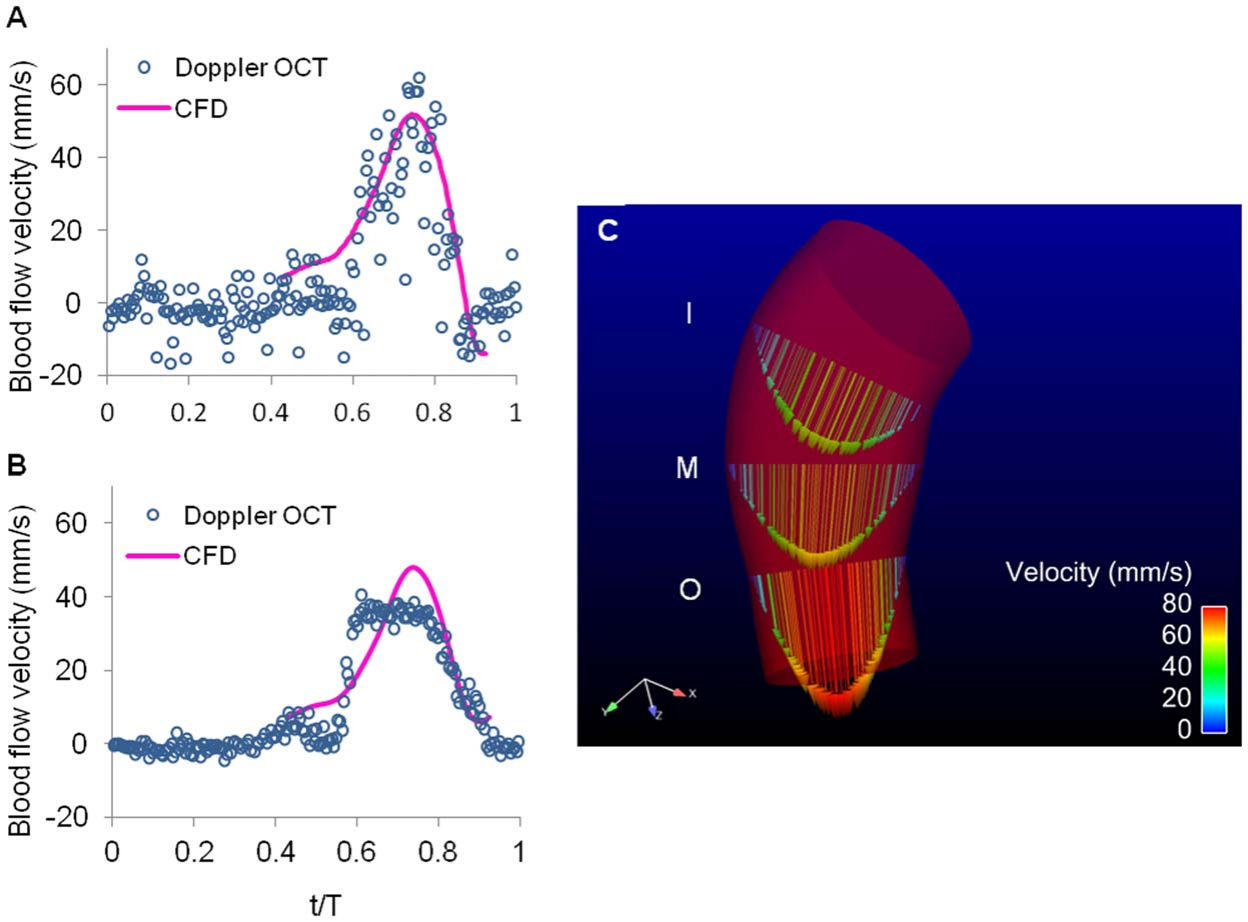
Blood flow dynamics within the OFT of the representative HH18 chick embryo. (A) and (B) Comparison of centerline velocities measured with Doppler OCT and calculated with our CFD model at approximately the centerline of cross-sectional planes 1 and 2, respectively (see Figure 1A). (C) CFD model of the heart OFT at maximal wall expansion, showing velocity profiles at three cross-sectional planes: I, M and O.

To further quantify blood flow within the OFT as well as wall shear stresses, we generated a subject-specific CFD model of the representative HH18 chick embryo OFT. Based on the analysis of Doppler OCT images (see Figure 8) showing there was no significant flow when lumen area was small, we simulated flow for about 50% of the cardiac cycle (∼0.43 T to ∼0.93 T, with T = 0.37 s, Video S2), spanning time points during which the lumen area increased, reached a maximum and then decreased. Note that this is different from our previously published CFD model of the OFT [33], in which the whole cycle was simulated. Centerline velocities along the OFT calculated from the CFD model were consistent with Doppler OCT measurements (Figures 9A and 9B).

**Figure 10:**
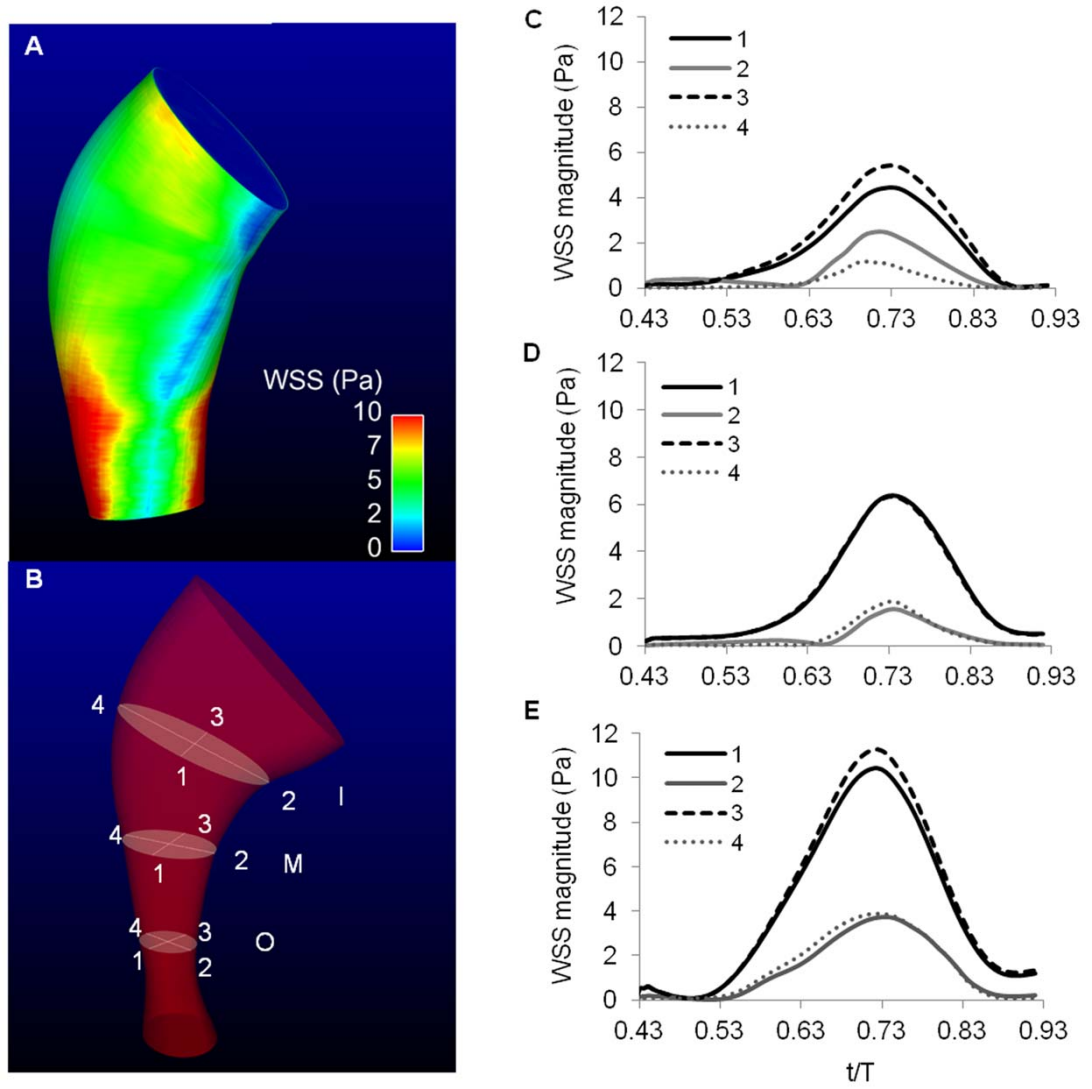
Wall shear stress distribution calculated from our CFD model of the OFT. (A) Spatial distribution of wall shear stress on the OFT endocardium surface when the OFT wall was fully expanded. (B) Sketch of the OFT model showing the cross-sectional planes (I, M, and O) that were used for further wall shear stress comparisons. For each plane, 4 points along the endocardium were analyzed: points 1 and 3 located on the surface of the cardiac cushions, and points 2 and 4 along the long axis of the elliptical lumen. (D), (D) and (E) Wall shear stress over time at the points (1 to 4 shown in B) on the cross-sectional planes I, M, and O, respectively. WSS: wall shear stress.

Blood flow through the OFT did not show re-circulation regions, nor significant backflow. Since the OFT lumen was slightly tapered distally, maximal velocities occurred at the distal region of the OFT (Figure 9C). Further, the OFT curvature resulted in blood flow velocities that were slightly skewed towards the OFT inner curvature (Figure 9C). The presence of cardiac cushions, which rendered an elliptical lumen cross-section, resulted in a flow distribution with maximal velocities around the center of the elliptical section, and larger velocity gradients in the direction of the ellipse minor axis, and a characteristic spatial distribution of wall shear stresses (Figure 10). Larger wall shear stresses were found on the endocardial cushion surfaces, and inner OFT curvature (Figure 10C), and maximal wall shear stresses on the endocardial cushion surfaces of the distal portion of the OFT. Wall shear stresses further exhibit a spiral pattern, following the orientation of the cushions along the OFT (Figure 10A). During the cardiac cycle, the largest wall shear stresses were found during fast blood flow ejection (see Figure 10 and Video S3).

### Uncertainty Quantification and Limitations

OFT motion was quantified from 4D OCT images of 7 chicken embryos. Variations in imaging results came from three main sources: (1) biological variations of the embryos, (2) experimental conditions, and (3) image quality and image processing procedures, including 4D image reconstruction and image segmentation. Biological variations of embryos are inevitable, and our analyses aim to characterize variations in cardiac OFT motion among 7 normal embryos. Efforts have been made to minimize variations in experimental conditions: (i) we tightly controlled temperature (within 1°C) during image acquisition, which resulted in changes of cardiac period during acquisition of only up to 7%; and (ii) we carefully examined embryos prior to and after image acquisition: embryos showing any signs of bleeding or anomalies were excluded. To ensure the accuracy of the 4D image reconstruction, we compared OFT wall dynamics from longitudinal OCT images extracted from the reconstructed images with those from direct OCT imaging. Phase (timing) errors in the 4D reconstructed images were estimated to be no more than 6%. We also estimated errors in image segmentation by comparing results from the automatic segmentation procedure against manual segmentation. We found that differences in calculated areas (enclosed by segmented boundaries) were no more than 10%. OCT image quality (signal to noise ratio) deteriorated with increased penetration depth, and affected image and image segmentations of the distal portion of the OFT (mainly cross-sectional planes 4 and 5, see Figure 1, and Video S1), which also explained the large variations observed in these regions (see Table 1). Overall, however, errors were relatively small, and 4D OCT imaging allowed us to study the motion of the chick heart OFT wall.

To simplify the analysis of the 4D reconstructed images, we analyzed 2D image sequences extracted from 5 cross-sectional planes along the OFT (planes 1 to 5, Figure 1). For these cross-sectional image sequences, the frame planes were fixed in space, and perpendicular to the OFT centerline when the OFT was most constricted. During the cardiac cycle, the motion of the heart slightly changes the position of the OFT centerline. This shift in the centerline, however, was calculated to be small (within 10 degrees) at any of the chosen planes, and thus the analyzed 2D planes were approximately perpendicular to the OFT centerline during the whole cardiac cycle. More importantly, by fixing the frame planes in space, the image sequences showed the combined effect of OFT expansion and contraction in both radial and longitudinal directions. This effect is more important in proximal OFT cross-sections, since distally the aortic sac is tethered. To better assess the effects of longitudinal OFT motions, we extracted cross-sectional images near the OFT inlet, but so that the planes of these cross-sectional images approximately followed the OFT longitudinal motion, estimated from the motion of the characteristic cardiac bend at the intersection between the ventricle and the OFT (see Video S1). Without correcting for the longitudinal motion, our analysis slightly overestimated the amplitude of the OFT wall motion (within 10%) with largest deviations occurring during maximal OFT expansion, when longitudinal stretch was also maximal. Since the motion of the cardiac walls employed in the CFD model of the OFT was quantified from lumen-wall segmentations from the selected cross-sectional planes (planes 1 to 5, Figure 1), these uncertainties affect CFD results. Further, uncertainties related to pressure boundary conditions, measured in different embryos due to experimental limitations, and incorporated as normal traction boundary conditions in the model could further affect computations. However, we expect the effect of these inaccuracies on blood flow dynamic simulations to be small, especially when flow is maximal, or close to maximal. This was further confirmed by comparison of blood flow velocities computed with the CFD model and measured with Doppler OCT (see Figure 9) Considering the nature of the images acquired, and the fast beating cycle of the developing heart, uncertainties in measurements and CFD modeling are not large, and certainly well within experimental uncertainties. Quantified values, therefore provide a good estimation of the actual strains and stresses in cardiac tissues.

## Discussion

Blood flow is essential for normal cardiac development [1], and biomechanical stimuli (cardiac wall stresses and strains) are known to affect heart development by modulating genetic programs [1], [39]. Despite the recognized importance of biomechanical stimuli on cardiac development, little is known about the biological mechanisms by which these stimuli influence cardiac growth and development, and that can lead to detrimental adaptations that result in congenital heart defects. This is partly due to a lack of reliable methods to quantify mechanical stimuli in the rapidly beating developing heart. Previous studies mainly focused on morphological and physiological changes, e.g. [4], [14], [21], and gene expression changes [22] after hemodynamic interventions. Other studies focused on quantifying cardiac motion [27], [40], [41] or flow and wall shear stress [28], [42], [43]. In this work, using an integrative strategy, we characterized cardiac wall motion and blood flow dynamics, as well as wall stresses and strains, in the OFT of HH18 chicken embryos.

The heart OFT at HH18 is very sensitive to hemodynamic conditions. At early developmental stages, the OFT acts as a primitive valve: during ventricular systole blood flows through the OFT to the arterial system, and during ventricular diastole OFT wall contraction prevents backflow. Proper function of the OFT is thus critical for cardiac function. The interaction between cardiac wall motion and blood flow in the OFT provides biomechanical stimuli to cardiac tissues, stimuli that determine the fate of the intraventricular septum and semilunar valves. The motion of the OFT walls, and the tissue strains and stresses that result from the interaction between OFT cardiac walls and blood flow dynamics have not been studied. Using a combination of 4D OCT imaging, physiological measurements, and CFD modeling, the *in vivo* interaction between cardiac wall motion and blood flow dynamics within the HH18 chick OFT was characterized. Together, these data provided the basis for quantifying biomechanical stimuli on cardiac cells in the normal HH18 chick embryonic OFT. These quantifications could later be used when assessing changes in biomechanical stimuli due to mechanical interventions, and the effects of those changes on cardiac development and in the formation of congenital heart defects.

### Cardiac Wall Motion

At early developmental stages, including HH18, the OFT wall consists of three layers (myocardium, cardiac jelly, endocardium). The properties of the wall layers and their interaction over the cardiac cycle contribute to a unique motion pattern in the OFT wall, which is critical to regulate blood flow when there is no valve between the heart ventricle and vasculature. The interaction between blood flow and cardiac tissues generates unique local biomechanical stimuli for cardiac cells. Interestingly, we found different OFT wall motion patterns proximally (close to the ventricle) than distally (close to the aortic sac).

Analyses of OCT images of the chick heart OFT at HH18 revealed a peristaltic-like cardiac wall motion (see Video S1), with the lumen closing proximally to distally after ventricular systole. However, while the proximal OFT actively contracts, its distal portion may simply follow intracardiac pressure changes. This is consistent with decreased myocardial contractility towards the distal OFT (see Figure 2B), and the observation that the ASF of the lumen and myocardium significantly decreased at cross-sectional plane 5 (Figure 6B). This is not entirely surprising, however, since the distal portion of the OFT is a transition region between the heart and the arterial pole. The OFT connects distally to the aortic sac, which, from optical images, seems to be tethered. Further, aortic sac walls are passive and thus do not actively contract. From OCT images, however, it is difficult to estimate the distance between the aortic sac and the imaged distal portion of the OFT. Recent studies showed that the entire OFT external layer exhibit myocardial phenotype at HH18 [44] and that from stage HH14 to HH20 cells that migrate from the secondary heart field to the distal OFT [45] (from the arterial pole) are induced to differentiate into OFT cardiomyocytes [46], [47]. Our results suggest that the lack or reduced active contractility of the distal OFT wall may be due to the presence of immature myocardial cells, a geometrical constraint on wall motion imposed by the (stiffer) aortic sac wall, or a combination of both.

The uneven distribution of cardiac jelly around the myocardium layer, forming a pair of endocardial cushions that face each other, greatly facilitates lumen opening and closure over the cardiac cycle. The elliptical lumen shape rendered by the endocardial cushions has been shown to be more efficient than a circular lumen shape not only to achieve full lumen closure but also to pump blood more effectively [48]. This is consistent with a larger ASF for the lumen than the myocardium or cardiac jelly (Figure 6B). Cardiac cushions, therefore, facilitate lumen closure while letting the lumen reach a large area (upon myocardial relaxation) that allows blood to flow from the ventricle to the arterial system.

Cardiac jelly area, which can be used as a measure of the jelly mass since the jelly is approximately incompressible, changed by approximately 30% over the cardiac cycle (Figure 6B). These variations in cardiac jelly mass over time could be the result of (1) longitudinal motions of cardiac jelly due to hemodynamic forces (blood pressure and shear stress); and (2) longitudinal cyclic stretch of the myocardium, which also stretches the jelly longitudinally. At HH18, the cardiac jelly in the OFT is mainly composed of glycosaminoglycans [49], which make the cardiac jelly a very soft material [40], [50]. Larger cardiac jelly areas mainly correlated with a closed lumen (Figures 3B and 3C), when blood pressure from the ventricle was low (Figure 7C). There was however a slight increase in cardiac jelly area when the myocardium started to relax, which might indicate lateral motion of the cardiac jelly. This motion allowed the lumen to remain closed even after myocardium relaxation started, and therefore to extend lumen closure. Upon lumen expansion, when blood pressure and wall shear stresses were high (Figures 7C–7D, and Figures 10C–10D), cardiac jelly area decreased (Figures 3B and 3C), possibly due to longitudinal cardiac jelly motion. The area changes exhibited by the cardiac jelly, however, can also be explained by the longitudinal stretch of the OFT, which is approximately in phase with radial wall expansion, and results in about 20% myocardial elongation. Cardiac jelly has to distribute along the stretched OFT length, resulting in a decrease of cardiac jelly area at a given cross-section. Accounting for inaccuracies in cardiac jelly area quantification (between 10% and 20%) the longitudinal stretch of the myocardium could account for most (or possibly all) of the changes in cardiac jelly mass observed over the cardiac cycle. Previous studies have shown the existence of fibrils (e.g. fibronectin, tenascin C) that are attached to the endocardium and myocardium layers through the cardiac jelly [35], [41]. Fibrils seemingly act as molecular tethers in regions in which the cardiac jelly thickness is minimal (and remains approximately constant over the cardiac cycle). OCT images showed that, upon myocardial contraction, the endocardium first contracts and then folds along lines of minimal cardiac jelly thickness. This is consistent with the existence of molecular tethers. Fibrils, therefore, seem to aid in achieving proper lumen closure, and presumably limit cardiac jelly motion.

At any cross-section, the area of the lumen reflects the interaction between blood flow dynamics and cardiac tissues. As the myocardium contracts (both longitudinally and circumferentially), endocardial cushion pairs become closer to each other, until they eventually close the lumen. Subsequent lumen expansion occurs due to myocardial relaxation and is also likely facilitated by the high pressure of blood ejecting from the ventricle. Since the OFT lumen closed sequentially (in a peristaltic-like fashion) proximally to distally (Figure 4C), overall the OFT lumen was closed for about half of the cardiac cycle period (∼0.5 T). This is consistent with Doppler Ultrasound [51] and Doppler OCT measurements, which showed lack of significant blood flow for approximately half of the cycle. The relatively long closure time of the OFT lumen suggests that the OFT functions as an effective valve, ensuring sufficient ventricular filling time while preventing backflow.

### Blood Flow Dynamics

Simultaneous M-mode and M-phase images obtained from OCT, revealed the interaction between cardiac wall motion and blood flow dynamics whithin the OFT (see Figure 8). These images showed an intermittent blood flow regulated by the cyclic expansion and contraction of the OFT wall. There is no significant flow of blood for approximately half of the cardiac cycle, and then blood flow velocities sharply increase, reach a maximum and then decrease when the OFT walls are expanding and then contracting. These results further confirm the valvular function of the OFT wall at early stages of cardiac development.

Doppler OCT could be potentially used to calculate blood flow velocities in the OFT in 4D. However, several difficulties inherent to Doppler OCT measurements prevent accurate quantifications of blood flow velocities in 4D. These difficulties include (see also [28]): i) phase noise; ii) limitations in the maximum speed that can be measured, which leads to phase wrapping and wash-out; iii) regions of flow that are perpendicular to the direction of the OCT light, which render no Doppler signal. Thus, at best, Doppler OCT can render blood flow velocities only at certain regions in the OFT. To overcome these difficulties we used a subject-specific CFD model of the OFT.

Our subject-specific CFD model of the OFT enabled quantification of the full 4D blood flow velocities within the lumen, and the distribution of wall shear stresses on the endocardium. We did not simulate blood-tissue interactions (also known as fluid-structure interaction) in the OFT, because of a lack of embryonic cardiac wall material properties, which are extremely difficult to measure accurately using current experimental techniques. Instead, we generated an image-based, subject-specific CFD model of the OFT, and imposed displacements obtained from 4D OCT imaging to the model lumen-wall surface. The motion of the cardiac walls employed in the CFD model of the OFT was quantified from lumen-wall segmentations from the selected cross-sectional planes (planes 1 to 5, Figure 1) from a representative embryo. The CFD model assumed no longitudinal motion of the OFT wall. We estimate that the longitudinal velocity of the OFT wall (when the wall is expanding or contracting) is 10∼100 orders of magnitude lower than the averaged axial velocity of blood flow. Thus the effects of longitudinal wall motion on blood flow, when the OFT is open, is negligible. In addition, lumen cross-sections were assumed to be elliptical in the CFD model. This assumption should not significantly affect results, as blood flow occurs when the OFT walls are open, and lumen cross-sections most closely resemble the shape of an ellipse (see Figure S1). Only when blood flow within the OFT is slow, that is at the beginning and end of our simulations, when the OFT walls are starting to open and then are almost closed, respectively, the longitudinal motion of the OFT wall and the elliptical cross-sectional assumption (as well as initial conditions) could significantly affect flow results. This time period, however, is estimated to be short (∼10% of our simulated results). Thus, except at the very beginning and end of the simulated time period, our CFD model of the OFT provides a good description of blood flow velocities within the OFT and wall shear stresses on the endocardium.

Comparisons of centerline blood flow velocities obtained from Doppler OCT measurements and velocities at corresponding locations from the CFD model (Figures 9A and 9B) were in good agreement. At the OFT inlet (corresponding to cross-sectional plane 1, see Figure 1), centerline OCT velocity data was noisy because of the large Doppler angle at the OFT inlet (θ = 84°), which amplified the noise (see Eq. 4). Nevertheless, Doppler OCT velocity data and CFD velocities agreed very well. Towards the OFT middle region (corresponding to cross-sectional plane 2, Figure 1), the CFD velocities and measured Doppler OCT velocities, were similar except in a region around peak centerline velocities. The blood flow velocity profile obtained from OCT measurements showed a plateau that was not observed in CFD quantifications. This plateau, however, was likely because V_z_ exceeded the OCT system maximal detectable range (±12 mm/s). After unwrapping the phase, the OCT system maximal detectable range was increased to 24 mm/s, and after correcting for the Doppler angle (θ = 58.4°) resulted in a maximal detectable velocity of ∼45 mm/s. When phase wrapping occurs, unwrapped velocities are less accurate than velocities captured before wrapping. Thus discrepancies between OCT and CFD velocities could be due to both inaccuracies in selecting the corresponding location of the points to compare, as well as less accurate velocity measurements due to wrapping. Despite this discrepancy, general consistency between blood flow velocities from CFD predictions and OCT measurements, in particular at the OFT inlet (plane 1), indicated that the velocity data calculated over time using our CFD model is fairly accurate.

While blood flow within the OFT was laminar (non-turbulent), blood flow inertia and the effect of the OFT wall motion on blood flow could not be completely neglected (maximum Reynolds number, a measure of the ratio of inertial to viscous forces, was ∼5). When comparing results obtained from CFD models in which inertial effects were fully considered, with results obtained under ‘quasi steady-state’ conditions in which inertial effects were neglected (and thus represent a series of ‘fixed’ geometries and steady-state flow), differences in flow were observed especially when blood volume flow rate was low (see Figure S3), especially at the beginning and end of calculations. The effect of wall motion on blood flow, however, was almost negligible during peak flow, when the OFT wall velocity is much smaller than the flow velocity. This comparison suggests that the motion patterns of the myocardial wall affect blood flow mainly during flow acceleration and deceleration phases. The distribution of blood flow, was also locally and globally affected by the distribution of endocardial cushions, and the curvature of the OFT.

### Biomechanical Stimuli on Cardiac OFT Walls

The interaction between cardiac tissue and blood flow dynamics determines the stresses and strains to which cardiac cells are subjected over the beating cycle. These biomechanical stimuli are known to modulate developmental cardiac genetic programs, and to be essential for proper cardiac development. Thus characterization of biomechanical stimuli on developing cardiac walls is essential for properly understanding the effect of biomechanics on cardiac development and ultimately how altered blood flow dynamics can lead to detrimental adaptations and heart defects.

#### a) Myocardium

During the cardiac cycle myocardial cells are subjected to large variations in cyclic strains. Contraction and expansion of the OFT myocardium occurred both in the circumferential direction (changes in radius) and longitudinal direction (changes in axial length). While strains were analyzed over cross-sections that were fixed in space, rather than moving with the cardiac tissue, and our approach could not resolve the residual strains (or morphogenic strains) that arise due to tissue growth [52], the quantified strains give a good estimation of strain variations (and thus mechanical stimuli variations) imposed on cardiac cells over the cardiac cycle. This estimation is likely very close to actual changes in strains, and could therefore be used as a basis for quantifications of biomechanical stimuli on OFT walls. From the most constricted to the most expanded configurations, the myocardium stretched between 20% and 40% in the circumferential direction (Figure 2C), and about 20% in the longitudinal direction. Our estimation of longitudinal stretch of the OFT myocardium agrees well with reported peak longitudinal strains in the primitive ventricle (0.13–0.16) at HH18 [53]. Further, our calculated circumferential strains were in agreement with reported circumferential fractional shortening of sarcomere spacing (0.40±0.04) in the myocardium of HH23 chick embryos [54]. Peak contraction circumferential strains at the proximal OFT (cross-sectional planes 1 to 4) were larger than estimated longitudinal strains, indicating that the OFT contracts primarily in the circumferential direction. In contrast, the distal OFT (cross-sectional plane 5) has a more isotropic strain pattern, with similar circumferential and longitudinal strains. Both circumferential strains and strain rates were significantly lower at the distal region of the OFT than at its proximal region (Figures 2C and 2D). This differential circumferential strain pattern follows the motion patterns of the myocardium layer (see Figures 2A and 2C), further suggesting that the distal OFT wall does not actively contract.

The motion of the OFT wall and its interaction with blood flow generates wall stresses in the myocardium. In the vasculature, circumferential wall stresses are generally uniform across the wall thickness, typically larger than radial stresses, and frequently estimated using the Laplace law. While the assumptions behind the derivation of the Laplace law do not entirely hold in the developing heart, it can nevertheless be used to ‘estimate’ the magnitude of wall stresses. In our calculations, we assumed that the myocardium bears the stress in the OFT wall and therefore we neglected stresses in the cardiac jelly, a very soft and incompressible material. Further, by using the Laplace law, we approximated the myocardium as a thin wall cylinder under steady state conditions. Like in the case of mature vascular walls, even though the OFT wall is not a thin-cylindrical structure, stresses along the tubular-heart wall radial direction are likely uniform (no changes in wall stress over the wall thickness), as a result of differential growth within the wall thickness [55]. Laplace law was therefore used to get a first approximation of the distribution of the wall stress along the OFT to enhance our understanding of mechanical stimuli on the OFT wall over the cardiac cycle. This first approximation, however, is likely representative of actual variations in wall stress in the heart OFT myocardium. Our results showed that wall stress in the myocardium increases towards the distal region of the OFT. This is because blood pressures along the OFT decreased only slightly, while the myocardium tapered and its thickness decreased distally (Figure S2). While this result needs further confirmation, it suggests that, during normal development, the OFT wall is subjected to a spatial (proximal to distal) gradient of wall stresses. Because wall stress has been proposed to be a critical biomechanical factor regulating myocardial proliferation in the embryonic heart [47], [56], this wall stress pattern may have important implications for normal cardiac development.

#### b) Endocardium

The endocardium, an endothelial cell layer lining the OFT lumen, is constantly exposed to cyclic strains imparted by deformations in response to blood flow and the active contraction of the myocardium, and to wall shear stresses induced by blood flow. Studies on mature vascular endothelial cells have shown that the interaction between wall shear stress and circumferential strain (their phase angle and magnitude) affect endothelial cell orientation, biochemical production, and gene expression [6], [57], [58]. Thus it is likely that in the developing heart both wall shear stresses and strains shape endocardial cell behavior, and cardiac development. Circumferential strains (Figure 5B) generally followed the deformation of the myocardium (Figure 2C), and drastically reduced distally (plane 5). Because the length of the endocardium in cross-sectional planes undergoes larger cyclic variations than the length of the myocardium, calculated circumferential strains were generally larger in the endocardium than the myocardium (Figure 6A). These large strains might be important for shaping the developing valves and septa, and proper orientation of endocardial cells and response to flow conditions.

Wall shear stresses on endocardial walls are heterogeneously distributed (see Figure 10). Wall shear stresses were higher on the cushion surfaces, inner curvature regions, and towards the distal regions of the OFT (where lumen area decreased). Changes in blood flow conditions (and thus wall shear stresses) have been shown to change the stiffness of cushions in the OFT and the expression of endothelial genes [22], [50]. The differential distribution of wall shear stresses on the OFT endocardium likely plays a pivotal role in organizing cells and extracellular matrix components in the endocardium and cardiac cushions, organization that is likely crucial for later septal and valve formation that originates in the OFT.

### Conclusions

Cardiac cells in the OFT are subjected to a combination of biomechanical stimuli (wall shear stress, strains, and wall stress). These biomechanical stimuli modulate genetic developmental programs and are needed for proper cardiac development: without flow, the heart does not develop properly, and thus hemodynamic stimuli are essential. The effect of biomechanical stimuli on cardiac development is even more obvious in developmental cardiac models of disturbed blood flow conditions, which lead to cardiac malformations. Despite the importance of biomechanics on cardiac development, biomechanical stimuli over cardiac developmental stages are not known due to limitations in current technologies. We proposed here an integrative methodology to bridge this gap and allow analyses of the effects of biomechanical stimuli on cardiac development and the pathways that lead to detrimental adaptations to hemodynamic conditions. Our study contributes to establishing and quantifying normal patterns of cardiac wall stress and strain in the developing heart, which will be of particular interest when analyzing deviations from normal patterns that lead to cardiac defects.

Our results show that the biomechanical environment to which cells are subjected varies over the cardiac cycle and over spatial locations within the OFT. Myocardial wall stresses increased distally, while myocardium and endocardium circumferential strains decreased distally, and wall shear stresses were maximal on the surface of cardiac cushions. This non-uniform distribution of biomechanical stimuli could give rise to regional-specific cellular responses and extracellular matrix organization within the OFT wall, and thus would almost certainly contribute to the extensive remodeling and morphogenetic events that occur in the OFT.

## Supporting Information

Figure S1 **Illustration of image processing on OCT images of the OFT.** (A), (B), and (C) Segmented contours of the OFT lumen (yellow), the interior myocardial boundary (green), and the exterior myocardial boundary (purple) overlaid on cross-sectional OCT images, and shown when the OFT is closed, opening, opened, respectively. (C), (E) and (F) Elliptical fits of the lumen (green curve) overlaid on the OCT images shown above. Scale bar = 200 µm.
(TIF)

Figure S2 **Calculated wall thickness of the myocardium at the 5 selected OFT cross-sections.** Wall thickness (h) was measured from OCT images of the representative embryo over the cardiac cycle. The plot shows the average value of the thickness over the cardiac cycle for each cross-sectional plane, as well as maximum and minimum values over the cardiac cycle.
(TIF)

Figure S3 **Effects of cardiac OFT wall motion on blood flow dynamics.** Comparisons of predicted centerline velocity profiles calculated from the CFD model, but assuming transient flow and quasi-steady flow in the OFT. (A), (B) and (C) Velocity profiles obtained from the centerline of cross-sectional planes I, M and O (see Figure 9C), respectively.
(TIF)

Video S1 **OCT images of the heart outflow tract (OFT) from the representative HH18 chick embryo.** The video of the longitudinal OFT section (on the left down corner) shows the wave-like motion of the myocardium, cardiac jelly and lumen along the OFT. The cross-sectional videos show the motion of the OFT at the 5 cross-sectional images along the OFT that were analyzed (see also Figure 1). M, myocardium; L, lumen; CJ, cardiac jelly. Scale bar = 200 µm.
(AVI)

Video S2 **Distribution of blood flow velocities in the HH18 chick OFT, calculated using the CFD model.** For easier visualization, blood flow profiles were depicted along the major axis of the elliptical cross-sectional lumen areas, at planes I, M, and O (see Figure 9C).
(MPG)

Video S3 **Distribution of wall shear stresses (WSS) on the OFT endocardium, calculated using the CFD model.** Wall shear stresses are depicted over the cardiac cycle.
(MPG)
