# Supplementary figures and images for: Biomechanics of the Chick Embryonic Heart Outflow Tract at HH18 Using 4D Optical Coherence Tomography Imaging and Computational Modeling
Source: PLoS One. 2012 Jul 23;7(7):e40869. doi: 10.1371/journal.pone.0040869 (PMC3402486; doi:10.1371/journal.pone.0040869)

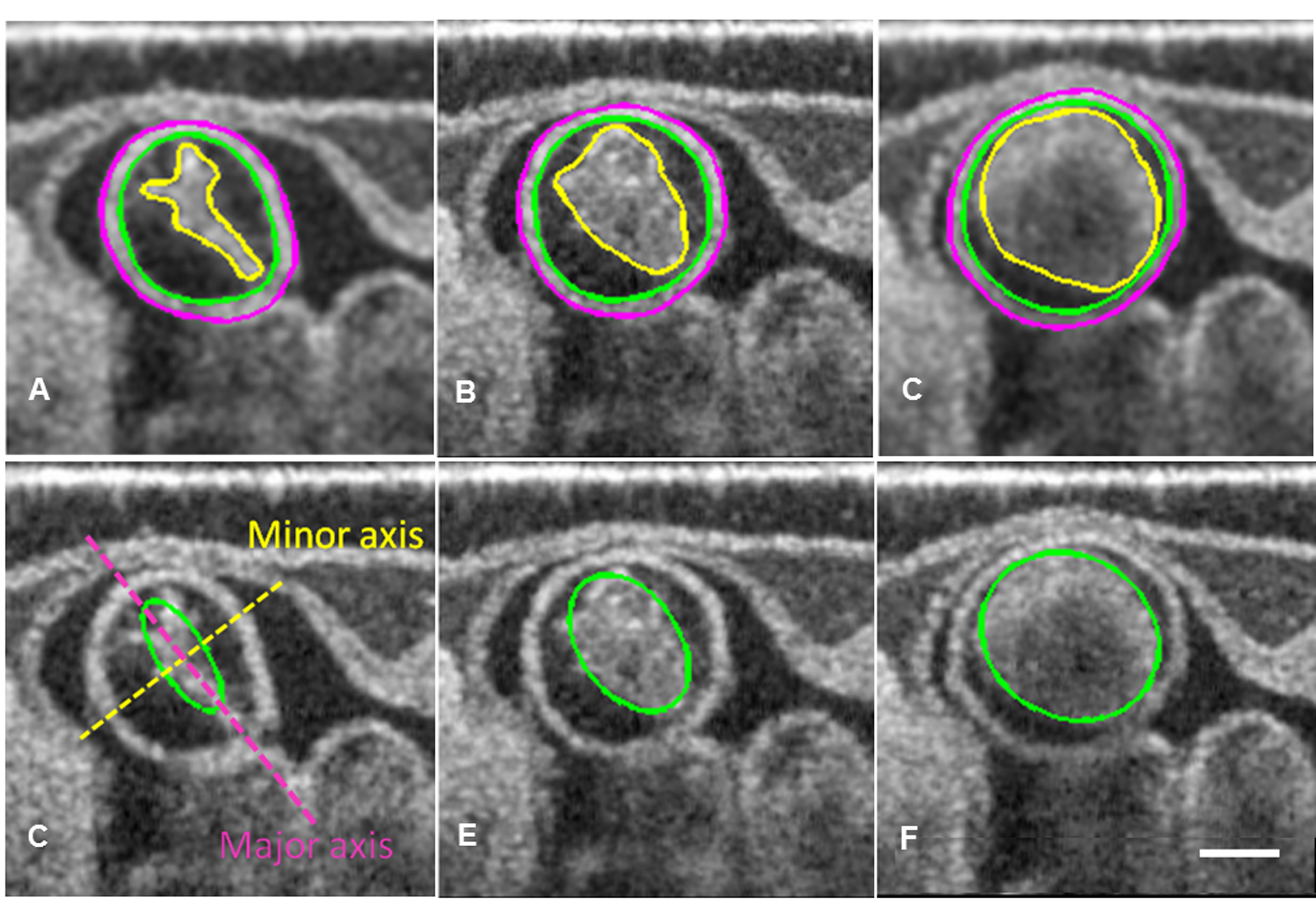

Supplement: Figure S1 — Illustration of image processing on OCT images of the OFT. (A), (B), and (C) Segmented contours of the OFT lumen (yellow), the interior myocardial boundary (green), and the exterior myocardial boundary (purple) overlaid on cross-sectional OCT images, and shown when the OFT is closed, opening, opened, respectively. (C), (E) and (F) Elliptical fits of the lumen (green curve) overlaid on the OCT images shown above. Scale bar = 200 µm. (TIF) [file pone.0040869.s001.tif]

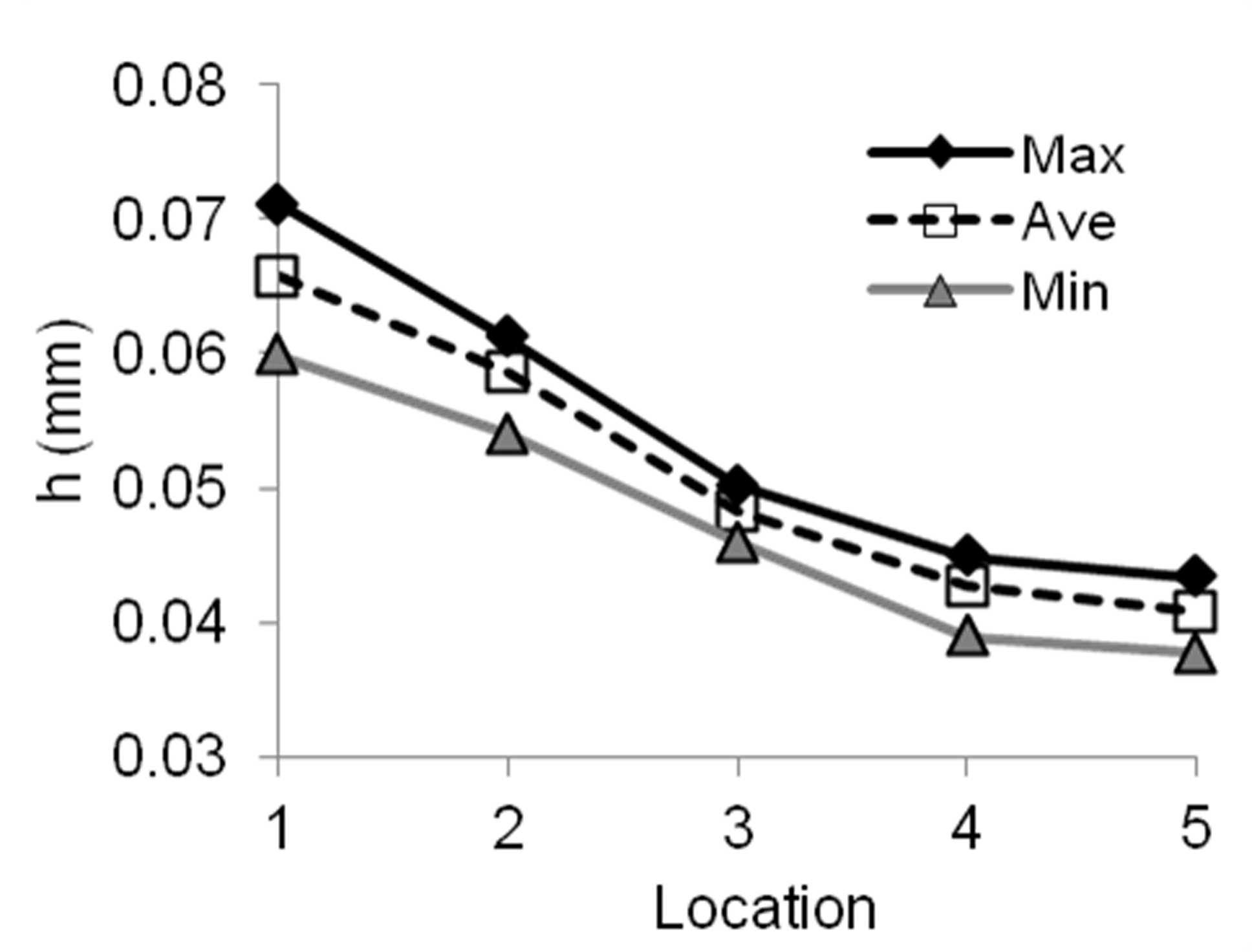

Supplement: Figure S2 — Calculated wall thickness of the myocardium at the 5 selected OFT cross-sections. Wall thickness (h) was measured from OCT images of the representative embryo over the cardiac cycle. The plot shows the average value of the thickness over the cardiac cycle for each cross-sectional plane, as well as maximum and minimum values over the cardiac cycle. (TIF) [file pone.0040869.s002.tif]

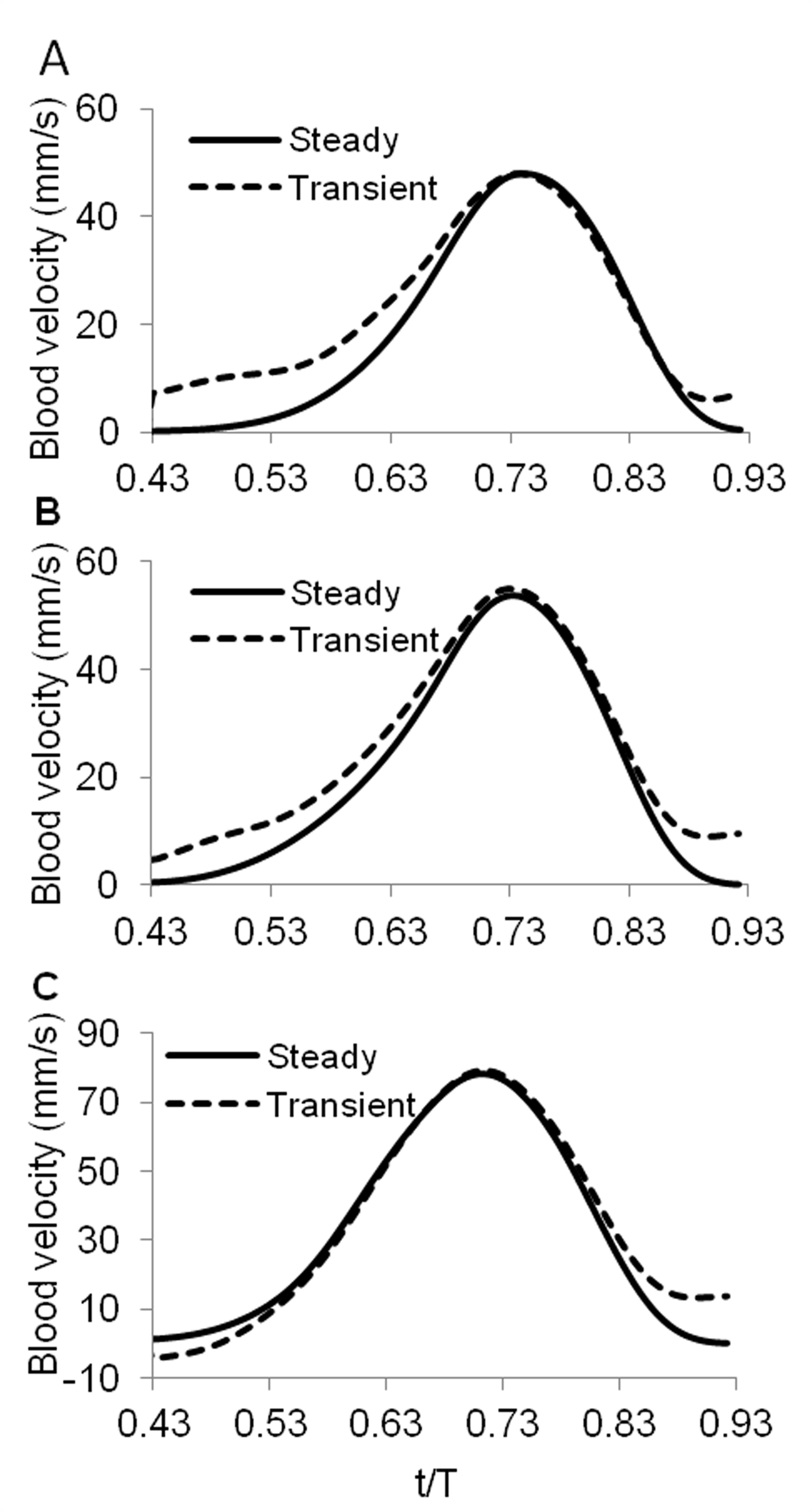

Supplement: Figure S3 — Effects of cardiac OFT wall motion on blood flow dynamics. Comparisons of predicted centerline velocity profiles calculated from the CFD model, but assuming transient flow and quasi-steady flow in the OFT. (A), (B) and (C) Velocity profiles obtained from the centerline of cross-sectional planes I, M and O (see Figure 9C), respectively. (TIF) [file pone.0040869.s003.tif]
